# The BET bromodomain inhibitor exerts the most potent synergistic anticancer effects with quinone-containing compounds and anti-microtubule drugs

**DOI:** 10.18632/oncotarget.12640

**Published:** 2016-10-13

**Authors:** Pei Y. Liu, Nicholas Sokolowski, Su T. Guo, Faraz Siddiqi, Bernard Atmadibrata, Thomas J. Telfer, Yuting Sun, Lihong Zhang, Denise Yu, Joshua Mccarroll, Bing Liu, Rui H. Yang, Xiang Y. Guo, Andrew E. Tee, Ken Itoh, Jenny Wang, Maria Kavallaris, Michelle Haber, Murray D. Norris, Belamy B. Cheung, Jennifer A. Byrne, David S. Ziegler, Glenn M. Marshall, Marcel E. Dinger, Rachel Codd, Xu D. Zhang, Tao Liu

**Affiliations:** ^1^ Children's Cancer Institute Australia for Medical Research, University of New South Wales, Sydney, Australia; ^2^ School of Biomedical Sciences and Pharmacy, University of Newcastle, Newcastle, Australia; ^3^ School of Medical Sciences (Pharmacology) and Bosch Institute, The University of Sydney, Sydney, Australia; ^4^ Department of Anatomy, Histology and Embryology, School of Basic Medical Sciences, Fudan University, Shanghai, China; ^5^ Department of Molecular Biology, Shanxi Cancer Hospital and Institute, Affiliated Hospital of Shanxi Medical University, Shanxi, China; ^6^ Department of Stress Response Science, Center for Advanced Medical Research, Hirosaki University Graduate School of Medicine, Hirosaki, Japan; ^7^ Centre for Childhood Cancer Research, University of New South Wales Medicine, University of New South Wales Australia, Sydney, Australia; ^8^ Children's Cancer Research Unit, Kids Research Institute, The Children's Hospital at Westmead, Westmead, Australia; ^9^ Kids Cancer Centre, Sydney Children's Hospital, High Street, Randwick, Australia; ^10^ Garvan Institute of Medical Research, Darlinghurst, Australia; ^11^ St Vincent's Clinical School, University of New South Wales Medicine, University of New South Wales Australia, Darlinghurst, Australia

**Keywords:** JQ1, neuroblastoma, quinone-containing compounds, vincristine, nanaomycin

## Abstract

BET bromodomain inhibitors are very promising novel anticancer agents, however, single therapy does not cause tumor regression in mice, suggesting the need for combination therapy. After screening a library of 2697 small molecule compounds, we found that two classes of compounds, the quinone-containing compounds such as nanaomycin and anti-microtubule drugs such as vincristine, exerted the best synergistic anticancer effects with the BET bromodomain inhibitor JQ1 in neuroblastoma cells. Mechanistically, the quinone-containing compound nanaomycin induced neuroblastoma cell death but also activated the Nrf2-antioxidant signaling pathway, and the BET bromodomain proteins BRD3 and BRD4 formed a protein complex with Nrf2. Treatment with JQ1 blocked the recruitment of Nrf2 to the antioxidant responsive elements at Nrf2 target gene promoters, and JQ1 exerted synergistic anticancer effects with nanaomycin by blocking the Nrf2-antioxidant signaling pathway. JQ1 and vincristine synergistically induced neuroblastoma cell cycle arrest at the G_2_/M phase, aberrant mitotic spindle assembly formation and apoptosis, but showed no effect on cell survival in normal non-malignant cells. Importantly, co-treatment with JQ1 and vincristine synergistically suppressed tumor progression in neuroblastoma-bearing mice. These results strongly suggest that patients treated with BET bromodomain inhibitors in clinical trials should be co-treated with vincristine.

## INTRODUCTION

Neuroblastoma is the most common extracranial solid tumor in children [1–3]. *MYCN* gene amplification occurs in a quarter of primary neuroblastomas and is associated with N-Myc protein over-expression and poor patient survival [4–6].

N-Myc belongs to the Myc family of oncoproteins, which induce cell proliferation, malignant transformation and tumor progression [7, 8]. Myc oncoproteins are considered “undruggable” because they have no active site for ligand binding by conventional small drug-like molecules and have not been crystalized [9, 10]. Thus, targeting factors upstream of Myc proteins provides an attractive approach for inhibiting the Myc oncogenic pathway. On the other hand, chemotherapeutic agents are currently used in the clinic at high doses, are often toxic to normal proliferating cells, and frequently lead to the development of drug resistance [11].

The BET bromodomain proteins, BRD3 and BRD4, bind to acetylated lysine residues on histones and regulate the expression of important oncogenes, such as *MYC*, *MYCN* and BCL2 [12–16]. JQ1 and I-BET151 are potent BET bromodomain inhibitors which interrupt the recruitment of BRD3 and BRD4 to chromatin to down-regulate *MYC*, *MYCN* and *BCL2* expression [12, 15–21].

Despite their promising therapeutic potential, BET bromodomain inhibitors do not cause tumor regression as single agents *in vivo* [12, 15–18, 20, 21]. We hypothesize that BET bromodomain inhibitors may be best utilized in combination with other anticancer agents to achieve the maximum effects against cancer cells and to minimize toxicity to normal cells.

In this study, chemical library screen identified quinone-containing compounds and anti-microtubule drugs as the two major groups of agents which exerted the best synergistic anticancer effects with JQ1. Vincristine was identified as the Food and Drug Authority (FDA)-approved drug which exerted the best synergistic anticancer effects with JQ1 *in vitro*, and combination therapy with JQ1 and vincristine was highly effective in suppressing tumor progression in neuroblastoma-bearing mice.

## RESULTS

### Small molecule library screen identifies two major groups of compounds that synergize with JQ1

**Figure 1:**
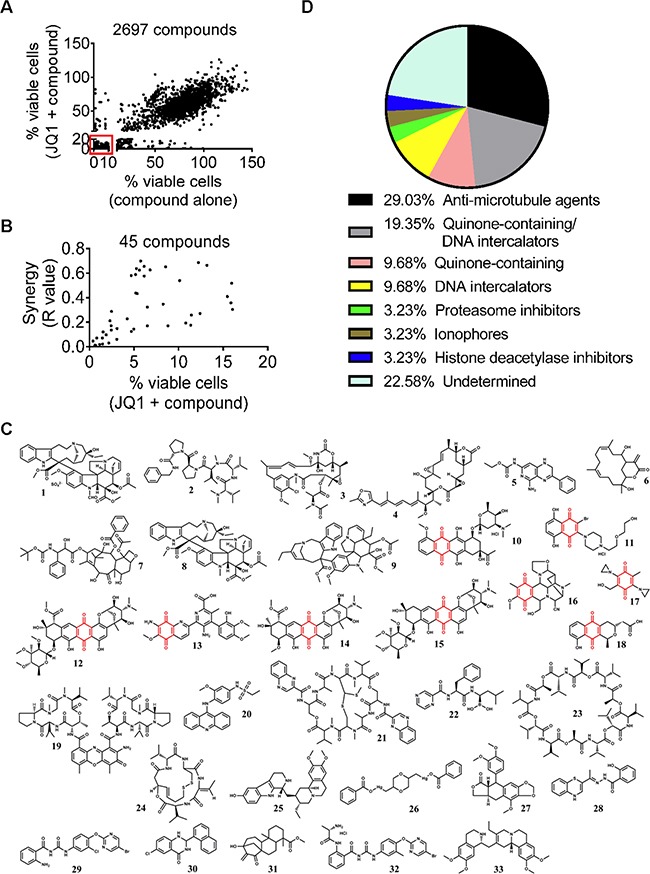
Small molecule library screen identifies two major groups of compounds that synergize with JQ1 **A.** BE(2)-C cells were treated with vehicle control, 200nM JQ1, 1μM or 10μM 2697 small molecule compounds or combination for 72 hours, followed by Alamar blue assays. A total of 273 compounds reduced the number of viable BE(2)-C cells by ≥ 90% on their own and ≥ 80% when combined with JQ1, as highlighted in the box. **B.** Forty-five potential “hit” compounds reduced the number of viable BE(2)-C cells by more than 80% when combined with 200nM JQ1 and have R values less than 0.7 (R<0.7). R values of <1, 1, or >1 indicated synergistic, additive and antagonistic effects respectively. **C.** Structures of the 33 compounds that exerted strong synergy with JQ1 from the secondary screen and numbers in bold correlated with entries in Table 1. The structure in red indicated quinone. **D.** “Hit” compounds were grouped according to their mechanisms of action and chemical structures.

To identify small molecule compounds which exerted synergistic anticancer effects with JQ1, we undertook a chemical library screen using four compound sets from the National Cancer Institute USA: Approved Oncology Drugs, Natural Product, Diversity and Mechanistic sets of small molecules. In total, 2697 compounds were used at 1μM or 10μM to treat human BE(2)-C neuroblastoma cells either alone or in combination with 200nM JQ1 which reduced the number of BE(2)-C cells by 20% on its own. Cytotoxicity testing using Alamar blue assays showed that 273 compounds reduced the number of viable BE(2)-C cells by ≥ 90% on their own, and also reduced the number of viable BE(2)-C cells by ≥ 80% when combined with JQ1 (Figure 1A and Supplementary Dataset S1). As these compounds showed high cytotoxicity on their own, it was not possible to calculate synergism of the combination treatments. When combined with JQ1, 45 out of the remaining 2424 compounds showed significant synergistic anticancer effects [R < 0.7, fractional product method [22] and combination therapies reduced the number of BE(2)-C cells by more than 80% (Figure 1B).

The 273 compounds which reduced cell viability by ≥ 90% on their own and the 45 potential “hit” compounds were then subjected to secondary screen with multiple doses of JQ1, small molecule compounds or in combination. Compounds were selected as “hit” compounds if the R values of their combination therapies with JQ1 were between 0.4 and 0.7 or < 0.4, indicative of synergy and strong synergy respectively. Using this criterion, 58 and 33 compounds exhibited synergistic (Supplementary Dataset S2) and strong synergistic (Supplementary Dataset S3) anticancer effects, respectively, against BE(2)-C neuroblastoma cells when combined with JQ1.

Within these 33 compounds which exhibited strong synergy with JQ1, two major groups could be identified based on their chemical structures and mechanisms of action (Figures 1C and 1D and Table 1). The first group with nine members contained a redox-active quinone unit [23]. Six of the nine compounds are also known as DNA intercalators. The second group of compounds with nine members had variable structures, but could be grouped according to their mechanisms of action as anti-microtubule compounds. In particular, vincristine was the FDA-approved agent which exerted the best synergistic anticancer effect with JQ1.

**Table 1:** Compounds which exert strong synergistic anticancer effects with JQ1

Number	NSC Number	R value	Mode of action	Name
1	67574	0.145	M	Vincristine sulfate
2	669356	0.243	M	Cemadotin
3	153858	0.246	M	Maysanine
4	332598	0.293	M	Rhizoxin
5	330770	0.306	M	(5-Amino-1,2-dihydro-3-phenylpyrido[3,4-b]pyrazin-7-yl)-carbamic acid ethyl ester
6	301684	0.311	M	Crassin (2,12-dihydroxy-14-oxabicyclo[11.3.1]heptadeca-4,8-dien-15-one)
7	628503	0.342	M	Docetaxel
8	49842	0.38	M	Vinblastine Sulfate Hydrate
9	608210	0.359	M	Vinorelbine tartrate
10	258812	0.061	Q; IC	N,N-Dimethyldaunomycin HCl
11	659999	0.094	Q; IC	2-Bromo-5,8-dihydroxy-3-[4-[2-(2-hydroxyethoxy)ethyl]-1-piperazinyl]-naphthalene-1,4-dione HCl
12	70845	0.111	Q; IC	Nogalamycin
13	83950	0.241	Q; IC	Streptonigrin
14	102815	0.34	Q; IC	7-O-Methylnogalarol
15	265450	0.395	Q; IC	Nogamycin
16	349644	0.31	Q	Cyanocycline A
17	697726	0.34	Q	RH-1 or 2,5-Diaziridinyl-3-(hydroxymethyl)-6-methyl-1,4-benzoquinone
18	267461	0.398	Q	Nanaomycin
19	3053	0.273	IC	Dactinomycin
20	243928	0.281	IC	N-[4-(9-acridinylamino)-3-methoxyphenyl]-ethanesulfonamide monomethanesulfonate
21	526417	0.337	IC	Levomycin
22	681239	0.2	PI	Bortezomib
23	122023	0.225	ION	Valinomycin
24	754143	0.29	HDACI	Romidepsin
25	131547	0.306	ND	Marckine
26	30916	0.311	ND	MP 317 or SKI23340 or 2,5-Bis[(benzoyloxymercuri)methyl]-P-dioxane
27	126727	0.316	ND	5′-Desmethoxy-β-peltatin-A methyl ether
28	635121	0.351	ND	N'-[(1Z)-1-(1,4-benzothiazin-2-ylidene)ethyl]-2-hydroxybenzohydrazide
29	639828	0.352	ND	Antineoplastic 639828
30	175636	0.362	ND	6-Chloro-2,3-dihydro-2-(1-naphthalenyl)-4(1H)-quinazolinone
31	620358	0.373	ND	Methyl 13-hydroxy-15-oxo-kaurenoate
32	654260	0.394	ND	2-[[(2S)-2-amino-1-oxopropyl]amino]-N-[[[4-[(5-bromo-2-pyrimidinyl)oxy]-3-methylphenyl]amino]carbonyl]-benzamide HCl
33	129414	0.396	ND	(+-)-2,3-Dehydroemetine 2HCl

Q, quinone-containing; IC, intercalator; M, antimicrotubule; PI, proteasome inhibitor; ION, ionophore; HDACI, histone deacetylase inhibitor; ND, not determined.

### Quinone-containing compounds exert considerable synergistic anticancer effects with JQ1

**Figure 2:**
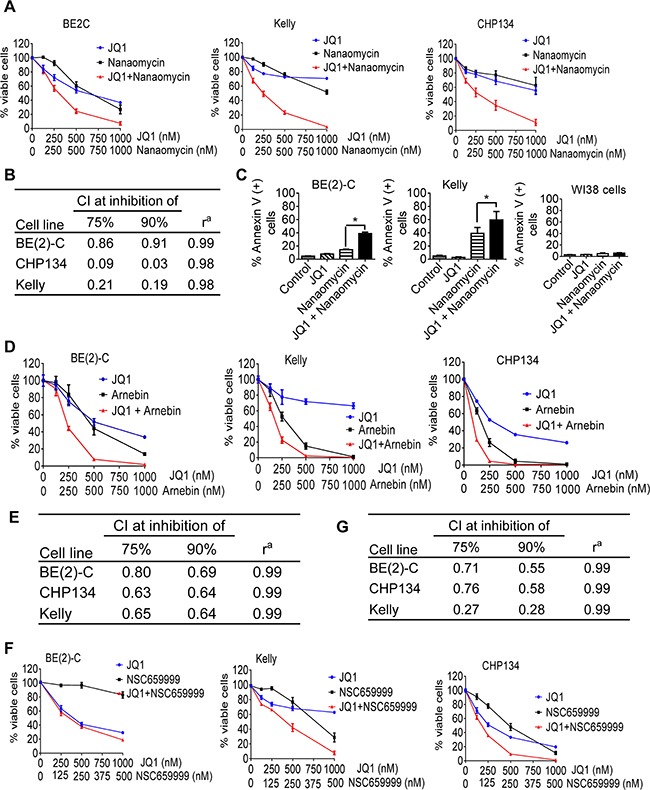
Quinone-containing compounds exert considerable synergistic anticancer effects with JQ1 **A.** BE(2)-C, Kelly and CHP134 neuroblastoma cells were treated with vehicle control, JQ1, nanaomycin, or combination at the indicated doses for 72 hours, followed by Alamar Blue assays. **B.** Combination indices (CIs) were generated for JQ1 plus nanaomycin combination therapy using the Calcusyn program; CI of <1, 1, or >1 indicates synergistic, additive and antagonistic effects respectively. CI_75%_ and CI_90%_ represented CIs for 75 and 90% reduction in the number of viable cells respectively. r^a^ is the linear correlation coefficient of the median-effect plot. **C.** BE(2)-C, Kelly and WI38 cells were treated with vehicle control, 500nM JQ1, 1000nM nanaomycin, or combination for 72 hours, followed by Annexin-V staining and flow cytometry analysis. **D-G.** BE(2)-C, Kelly and CHP134 neuroblastoma cells were treated with vehicle control, JQ1, arnebin, JQ1 plus arnebin (D and E), NSC659999, or JQ1 plus NSC659999 (F and G) at the indicated doses for 72 hours, followed by Alamar Blue assays (D and F). CIs were generated for combination therapy with JQ1 plus arnebin (E) or JQ1 plus NSC659999 (G). Error bars represent standard error. * indicates *P* < .05.

We examined whether quinone-containing compounds and JQ1 exerted synergistic anticancer effects in a range of neuroblastoma cell lines. Because the quinone-containing compound nanaomycin has been shown to function as a DNA methyltransferase 3B inhibitor [24, 25], we chose nanaomycin as the representative quinone-containing compound for mechanistic studies. Alamar blue assays showed that JQ1 and nanaomycin synergistically reduced the number of viable BE(2)-C, Kelly and CHP134 neuroblastoma cells (Figures 2A and 2B), but not WI38 embryonic fibroblasts (Supplementary Figure S1A). Flow cytometry analysis of Annexin-V positively stained apoptotic cells showed that JQ1 and nanaomycin synergistically induced apoptosis in 39% BE(2)-C (R = 0.64, fractional product method) and 59% Kelly (R = 0.37, fractional product method) neuroblastoma but not WI38 fibroblast cells (< 6%) (Figure 2C and Supplementary Figure S2). In addition, Alamar blue assays showed that JQ1 and nanaomycin synergistically reduced the number of viable c-Myc over-expressing PSN1 pancreatic, COLO320 and HCT116 colon cancer cells (Supplementary Figure S1B and S1C).

We next treated BE(2)-C, Kelly and CHP134 cells with a range of doses of JQ1 and/or the quinone-containing arnebin (Supplementary Dataset S2) or the quinone-containing/DNA intercalator compound 2-bromo-5,8-dihydroxy-3-[4-[2-(2-hydroxyethoxy)ethyl]-1-piperazinyl]-naphthalene-1,4-dione HCl (NSC659999) (Supplementary Dataset S3). Alamar blue assays showed that combination therapy with JQ1 and arnebin (Figures 2D and 2E) or compound NSC659999 (Figures 2F and 2G) synergistically reduced the number of viable BE(2)-C, Kelly and CHP134 cells, more substantial at medium doses than at high doses for arnebin due to strong effect from single therapy at high doses. Taken together, the data confirm that quinone-containing compounds including nanaomycin and arnebin, and quinone-containing/DNA intercalator compounds such as NSC659999 exert synergistic anticancer effects with JQ1 against neuroblastoma cells.

### JQ1 exerts synergistic anticancer effects with nanaomycin by blocking Nrf2-mediated antioxidant gene transcription

JQ1 exerts anticancer effects partly through blocking *MYCN* and *MYC* gene transcription [12, 20, 26]. To examine whether a reduction in N-Myc expression played a role in the synergistic anticancer effects of the combination of JQ1 and nanaomycin, we transfected *MYCN* gene amplified BE(2)-C and Kelly cells with control or N-Myc siRNAs, followed by treatment with a range of doses of nanaomycin. The N-Myc siRNAs had been shown to knock-down N-Myc gene expression previously [27]. Alamar blue assays showed that N-Myc siRNAs and nanaomycin did not exert synergistic anticancer effects (Supplementary Figure S3A), suggesting that reduction in N-Myc expression is not responsible for the synergistic anticancer effects of JQ1 and nanaomycin combination therapy.

Nanaomycin has been shown to function as a specific inhibitor of DNA methyltransferase 3B (DNMT3B) [24, 25]. We next transfected BE(2)-C and Kelly cells with control siRNA, DNMT3B siRNA-1 or DNMT3B siRNA-2, followed by treatment with JQ1. RT-PCR analysis showed that DNMT3B siRNAs reduced DNMT3B expression (Supplementary Figure S3B), and Alamar blue assays showed that DNMT3B siRNAs and JQ1 apparently did not exert synergistic anticancer effects (Supplementary Figure S3C). Moreover, combination therapy with JQ1 and the pan-DNMT inhibitor 5-Azacytidine or 5-Aza-2-deoxycytidine did not exert synergistic anticancer effects in BE(2)-C and Kelly cells (Supplementary Figures S3D and S3E).

**Figure 3:**
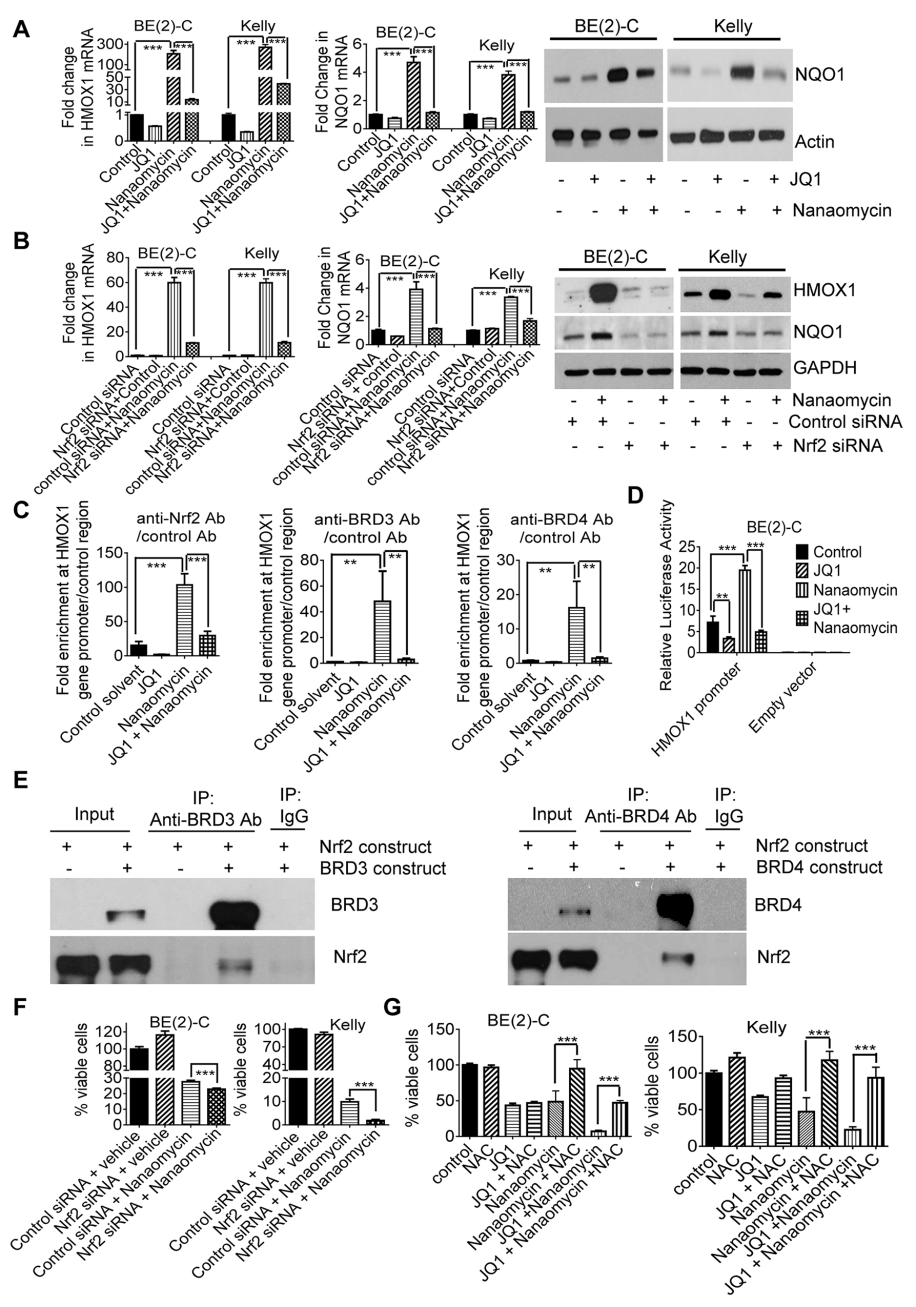
JQ1 exerts synergistic anticancer effects with nanaomycin by blocking Nrf2-mediated antioxidant gene transcription **A.** BE(2)-C and Kelly cells were treated with vehicle control, 500nM JQ1, 1000nM nanaomycin, or combination for 48 hours, followed by RT-PCR and immunoblot analyses of HMOX1 and NQO1 expression. **B.** BE(2)-C and Kelly cells were transfected with control or Nrf2 siRNA and treated with vehicle control or 1000nM nanaomycin for 48 hours, followed by RT-PCR and immunoblot analyses of HMOX1 and NQO1 expression. **C.** BE(2)-C cells were treated with vehicle control, 500nM JQ1, 1000nM nanaomycin, or combination for six hours, followed by ChIP assays and PCR with primers targeting the *HMOX1* gene core promoter or a negative control region. **D.** BE(2)-C cells were transfected with a luciferase reporter construct expressing the *HMOX1* gene promoter or empty vector, then treated with vehicle control, 500nM JQ1, 1000nM nanaomycin, or combination for 48 hours followed by luciferase assays. **E.** HEK-293 cells were co-transfected with a construct expressing Nrf2, together with a construct expressing empty vector, BRD3 or BRD4. Protein from the cells was immunoprecipitated (IP) with a control IgG, anti-BRD3 or anti-BRD4 antibody and analyzed by immunoblot. **F.** BE(2)-C and Kelly cells were transfected with control or Nrf2 siRNA and treated with vehicle control or 1000nM nanaomycin for 72 hours, followed by Alamar blue assays. **G.** BE(2)-C and Kelly cells were co-treated with vehicle control or 10mM NAC, together with vehicle control, 500nM JQ1, 1000nM nanaomycin or combination for 72 hours, followed by Alamar blue assays. Error bars represented standard error. *, ** and *** indicated *P* < .05, .01 and .001 respectively.

To identify the mechanism through which JQ1 and nanaomycin synergistically induced cell death, we performed differential gene expression studies with Affymetrix microarray in BE(2)-C cells six hours after treatment with vehicle control, JQ1, nanaomycin, or combination. The microarray data showed that treatment with nanaomycin considerably induced the expression of six genes *HMOX1*, *NQO1*, *AKR1C1*, *AKR1C2*, *GCLM* and *PPT2*, and that co-treatment with JQ1 blocked the effect (Supplementary Table S1). RT-PCR and immunoblot analyses confirmed that nanaomycin up-regulated *HMOX1* and *NQO1* expression in BE(2)-C and Kelly cells, and JQ1 largely blocked *HMOX1* and *NQO1* up-regulation (Figure 3A).

The transcription factor Nrf2 is well-known to activate the expression of *HMOX1*, *NQO1*, *AKR1C1*, *AKR1C2* and *GCLM* by binding to antioxidant responsive elements at the gene promoters [28]. We next transfected BE(2)-C and Kelly cells with control or Nrf2 siRNA, followed by treatment with control or nanaomycin. RT-PCR and immunoblot analyses confirmed that Nrf2 siRNA knocked-down *Nrf2* expression (Supplementary Figure S4A), and blocked nanaomycin-mediated *HMOX1* and *NQO1* up-regulation (Figure 3B). The data suggest that nanaomycin activates *HMOX1* and *NQO1* gene expression through Nrf2.

We then treated BE(2)-C and Kelly cells with control, JQ1, the quinone-containing arnebin, the quinone-containing/DNA intercalator compound NSC659999, combination of JQ1 and arnebin or compound NSC659999. RT-PCR showed that arnebin and compound NSC659999 considerably increased *HMOX1* and *NQO1* gene expression, and that JQ1 blocked or reduced the effect (Supplementary Figures S4B and S4C). The data suggest that quinone-containing compounds and quinone-containing/DNA intercalator compounds commonly induce Nrf2 pathway activation and that JQ1 blocks or reduces this effect.

We next examined whether nanaomycin induced the recruitment of Nrf2, BRD3 and BRD4 proteins to the Nrf2 target gene promoters. Chromatin immunoprecipitation (ChIP) assays revealed that treatment with nanaomycin led to significant enrichment of Nrf2, BRD3 and BRD4 proteins at the *HMOX1* gene promoter (Figure 3C), and that co-treatment with JQ1 blocked the effect (Figure 3C). Luciferase assays confirmed that treatment with JQ1 reduced, while treatment with nanaomycin enhanced, *HMOX1* gene promoter activity, and that co-treatment with JQ1 blocked nanomycin-mediated *HMOX1* gene promoter activity (Figure 3D). In addition, protein co-immunoprecipitation assays showed that an anti-BRD3 and an anti-BRD4 antibodies both efficiently co-immunoprecipitated Nrf2 protein (Figure 3E), demonstrating that both BRD3 and BRD4 proteins form a complex with Nrf2.

Nrf2 renders cancer cells resistant to oxidative stress-mediated cell death by activating the transcription of antioxidant genes including *HMOX1* and *NQO1* [29]. As nanaomycin activates Nrf2 signaling pathway, we transfected BE(2)-C and Kelly cells with control or Nrf2 siRNA and treated the cells with vehicle control or nanaomycin. Alamar blue assays showed that knocking down Nrf2 enhanced nanaomycin-mediated reduction in the number of viable neuroblastoma cells (Figure 3F). We next co-treated BE(2)-C and Kelly cells with the antioxidant N-acetylcysteine (NAC) or vehicle control, together with JQ1 and/or nanaomycin. Alamar blue assays showed that NAC largely reversed the reduction in the number of viable neuroblastoma cells after treatment with nanaomycin alone or JQ1 plus nanaomycin (Figure 3G), suggesting that Nrf2-antioxidant signaling pathway activation renders neuroblastoma cells insensitivity to nanaomycin treatment.

Taken together, the data suggest that (i) nanaomycin induces neuroblastoma cell death but results in Nrf2, BRD3 and BRD4 protein recruitment to the Nrf2 target gene promoters and increases promoter activity, leading to Nrf2 target gene up-regulation; (ii) JQ1 blocks nanaomycin-mediated Nrf2 target gene expression; and (iii) JQ1 exerts synergistic anticancer effects with nanaomycin by blocking Nrf2-antioxidant signaling pathway activation.

### The anti-microtubule drug vincristine exerts considerable synergistic anticancer effects with JQ1 against neuroblastoma cells

**Figure 4:**
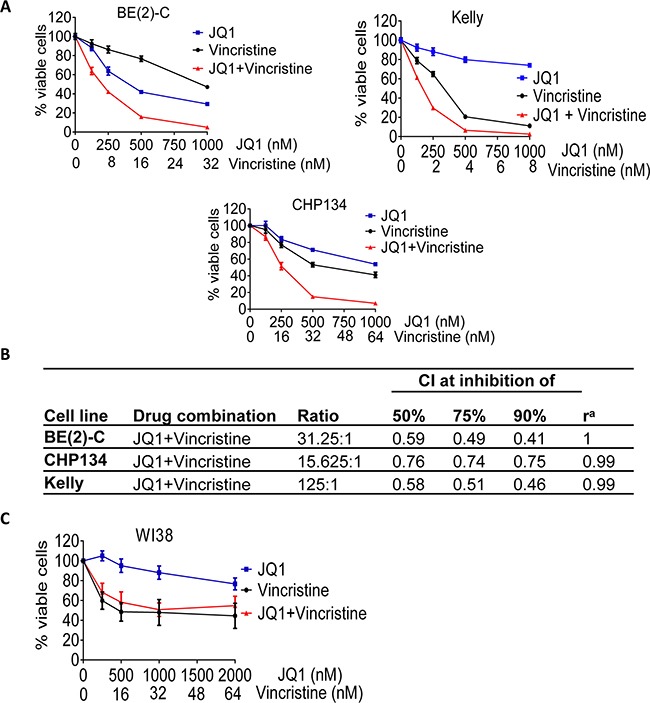
The anti-microtubule drug vincristine exerts considerable synergistic anticancer effects with JQ1 **A-B.** BE(2)-C, Kelly and CHP134 neuroblastoma cells were treated with vehicle control, JQ1, vincristine or combination at the indicated doses for 72 hours, followed by Alamar Blue assays. (B) CIs were generated for combination experiments with JQ1 plus vincristine. **C.** WI38 embryonic fibroblasts were treated with vehicle control, JQ1, vincristine or combination at the indicated doses for 72 hours, followed by Alamar Blue assays. Error bar represented standard error.

Our secondary screen showed that the other group of compounds which exerted synergistic anticancer effects with JQ1 in BE(2)-C cells was the anti-microtubule drugs, and vincristine exerted the best synergistic anticancer effect with JQ1 (Table 1 and Supplementary Dataset S3). We next treated BE(2)-C, Kelly and CHP134 neuroblastoma cells and WI38 fibroblasts with vehicle control, a range of doses of JQ1, vincristine, or combination. Alamar blue assays showed that JQ1 and vincristine synergistically reduced the number of viable BE(2)-C, Kelly and CHP134 neuroblastoma cells (CI ≤ 0.76 for all cases) (Figures 4A and 4B), but showed the same effect as vincristine alone on WI38 human embryonic fibroblast cells (Figure 4C). The data suggest that JQ1 and vincristine exert synergistic anticancer effects against a range of neuroblastoma cell lines with little toxicity against normal non-malignant cells.

### JQ1 and vincristine synergistically induce neuroblastoma cell cycle arrest at the G2/M phase, aberrant mitotic spindle formation and apoptosis

**Figure 5:**
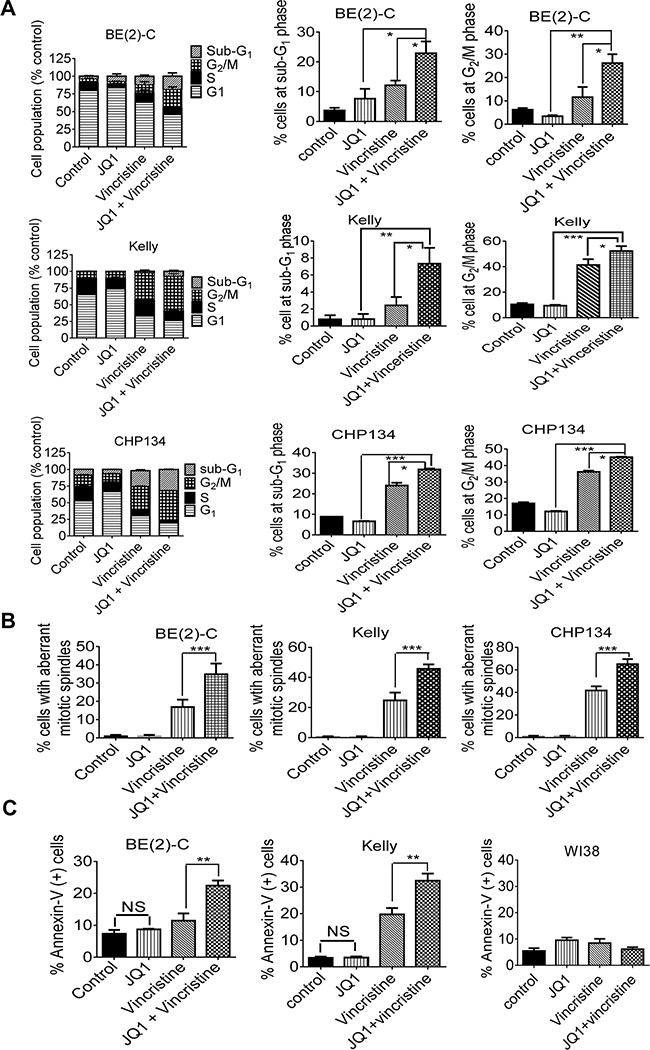
JQ1 and vincristine synergistically induce neuroblastoma cell cycle arrest at the G2/M phase, aberrant mitotic spindle formation and apoptosis **A** and **B.** BE(2)-C, Kelly and CHP134 neuroblastoma cells were treated with vehicle control, 500nM JQ1 and/or 8nM vincristine for 24 hours, followed by propidium iodide staining and flow cytometry analysis (A) or immunofluorescence staining (B) with an anti-α-tubulin antibody. DNA was counterstained with DAPI. Aberrant mitotic spindles were quantified by counting 50 cells per treatment group. **C.** BE(2)-C, Kelly and WI38 cells were treated with vehicle control, 500nM JQ1 (1000nM for Kelly and WI38 cells) and/or 8nM vincristine (16nM for WI38) for 24 hours, followed by staining with fluorescein isothiocyanate-conjugated Annexin-V and flow cytometry analysis. Error bar represented standard error. * indicated *P* < 0.05, ** *P* < 0.01, *** *P* < 0.001, and NS not significant.

Anti-microtubule drugs exert anticancer effects by destabilizing microtubules, inhibiting mitosis and inducing cell cycle arrest at the G_2_/M phase [30], and BRD4 is known to promote mitotic progression by designating genes for timely activation at the end of mitosis and at the early G_1_ phase [31]. We therefore performed cell cycle analysis, immunofluorescence study of α-tubulin and cell death analysis in BE(2)-C, CHP134 and/or Kelly cells after treatment with vehicle control, JQ1, vincristine, or combination. Cell cycle analysis showed that vincristine alone increased the proportion of neuroblastoma cells at the G_2_/M and sub-G_1_ phases, that JQ1 alone did not show the same trend, and that combination treatment with JQ1 and vincristine synergistically increased the proportion of neuroblastoma cells at the G_2_/M and sub-G_1_ phases (Figure 5A and Supplementary Figure S5). Immunocytochemistry revealed that vincristine alone increased the proportion of neuroblastoma cells with aberrant mitotic spindles, that JQ1 alone had no effect, and that combination treatment with JQ1 and vincristine synergistically increased the proportion of neuroblastoma cells with aberrant mitotic spindles (Figure 5B and Supplementary Figure S6). Flow cytometry analysis showed that JQ1 alone did not have an effect, that vincristine alone slightly increased the proportion of neuroblastoma cells positively stained by the apoptosis marker Annexin-V, and that JQ1 and vincristine synergistically and significantly increased the proportion of BE(2)-C and Kelly neuroblastoma, but not WI38 embryonic fibroblast (<10%), cells positively stained by Annexin-V (Figure 5C and Supplementary Figure S7). Taken together, the data suggest that JQ1 and vincristine synergistically destabilize microtubules, inhibit mitosis, induce cell cycle arrest at the G_2_/M phase and induce apoptosis in neuroblastoma but not normal non-malignant cells.

### JQ1 and vincristine synergistically suppress neuroblastoma progression in neuroblastoma-bearing mice

**Figure 6:**
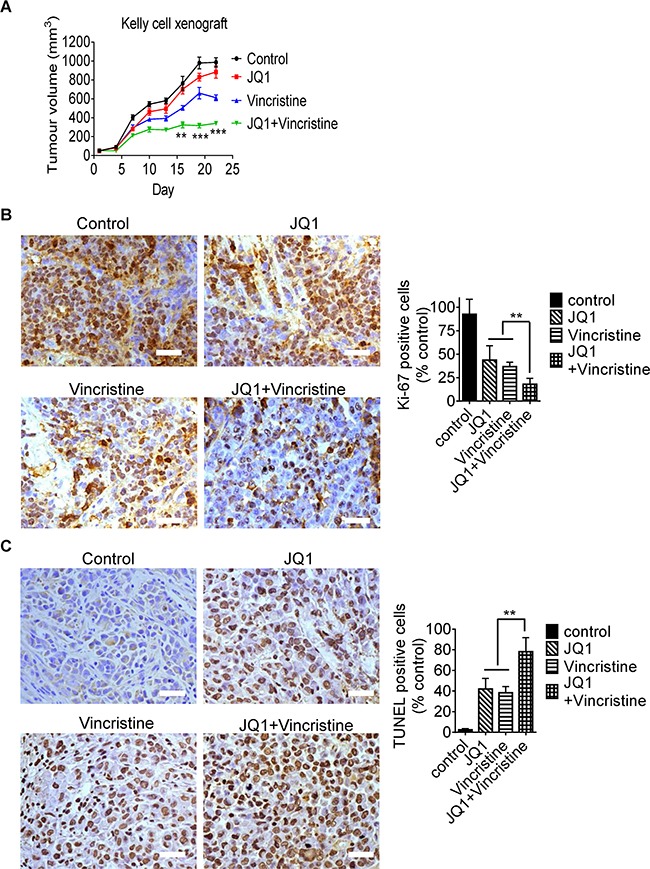
JQ1 and vincristine synergistically suppress neuroblastoma progression in neuroblastoma-bearing mice **A.** Nude mice were xenografted with Kelly neuroblastoma cells. When tumors reached 0.05cm^3^, mice were divided into four groups, and treated with vehicle control, JQ1 (50 mg/kg/day), vincristine (0.2 mg/kg every second day) or combination by intraperitoneal injection for three weeks. Tumor size was measured once every three days. **B** and **C.** Tumor tissues from the mice were immunostained with an anti-Ki-67 antibody (B) or TUNEL reagent (C) and visualized with diaminobenzidine (brown). The nuclei were counterstained with hematoxylin (blue). Cells positively stained by the anti-Ki67 antibody or TUNEL reagent were quantified and expressed as a percentage of control. Scale bars represent 100μm. ANOVA was used to calculate statistical difference. Error bars represent standard deviation. *** indicates *P* < 0.001 and ** *P* < 0.01.

Lastly, we examined whether JQ1 and vincristine exerted synergistic anticancer effects *in vivo*. Kelly neuroblastoma cells were xenografted into nude mice. Neuroblastoma-bearing mice were treated with vehicle control, JQ1, vincristine, or combination for 3 weeks. As shown in Figure 6A, treatment with JQ1 or vincristine inhibited neuroblastoma growth, compared with control, and combination therapy with JQ1 and vincristine synergistically reduced tumor growth (R = 0.70, 0.57 and 0.62 respectively for 16, 19 and 22 days of treatment, fractional product method). In addition, immunohistochemistry analysis showed that JQ1 and vincristine co-operatively reduced the proportion of neuroblastoma cells positively stained by the antibody against Ki-67 (Figure 6B), a marker for cell proliferation, and co-operatively increased the proportion of tumor cells positively stained by TUNEL (Figure 6C), a marker for apoptosis. Taken together, the data confirm that JQ1 and vincristine synergistically induce neuroblastoma cell growth inhibition and apoptosis in mice, and synergistically suppress the growth of established neuroblastoma tumors *in vivo*.

## DISCUSSION

BET bromodomain inhibitors are currently in multiple clinical trials in cancer patients. However, these inhibitors do not cause complete tumor regression as single agents in mouse models of cancers [16–18]. In the last two years, combination therapies with BET bromodomain inhibitors and other anticancer agents have been reported to achieve better anticancer efficacy than single therapies. For example, JQ1 and histone deacetylase inhibitors exert synergistic anticancer effects against pancreatic cancer [32], mantle cell lymphoma [33] and Myc-induced murine lymphoma [34] both *in vitro* and *in vivo*, and JQ1 in combination with the CDK inhibitor flavopiridol or the mTOR inhibitor rapamycin synergistically induces osteosarcoma cell apoptosis [35, 36]. However, it is unknown which anticancer agents should be combined with BET bromodomain inhibitors to achieve the best synergistic anticancer effects.

In this study, we used compound library screening to identify compounds that acted synergistically with JQ1 to reduce neuroblastoma cell viability. The histone deacetylase inhibitor romidepsin showed medium synergy when combined with JQ1, but the histone deacetylase inhibitor vorinostat, CDK inhibitors and rapamycin did not show clear synergy when combined with JQ1 in neuroblastoma cells. By contrast, two major groups of compounds have been identified from a library of 2697, based on chemical structures and mechanisms of action. One group consists of nine members with a redox-active quinone unit, and six of which are also known DNA intercalators. The other group contains nine well-characterized anti-microtubule agents. In particular, vincristine has been found to be the FDA-approved agent that exerts the most potent synergistic anticancer effects with JQ1.

The transcription factor Nrf2 binds to antioxidant responsive elements at the promoters of its target genes, such as *HMOX1* and *NQO1,* to activate antioxidant gene expression, and renders cancer cells resistant to oxidative stress-mediated cell death [28, 29]. Strikingly, our genome-wide gene expression study reveals that only six genes, including *HMOX1* and *NQO1*, are differentially expressed between nanaomycin and vehicle control treated cells, that the expression of the six genes is significantly reduced by JQ1 co-treatment, and that 5/6 genes are well-known Nrf2 pathway target genes. Protein co-IP, ChIP and luciferase assays show that Nrf2 forms protein complexes with BRD3 and BRD4, that treatment with nanaomycin increase Nrf2, BRD3 and BRD4 protein binding to the *HMOX1* gene promoter and increase *HMOX1* promoter activity, and that co-treatment with JQ1 suppresses the binding of Nrf2, BRD3 and BRD4 to the *HMOX1* gene promoter and *HMOX1* promoter activity. Importantly, knocking-down Nrf2 expression with siRNA, similar to treatment with JQ1, enhances nanaomycin-mediated cytotoxicity, and treatment with the antioxidant NAC largely block cell cytotoxicity induce by JQ1 and nanaomycin combination therapy. In addition, treatment with the quinone-containing compound arnebin or the quinone-containing/DNA intercalator compound NSC659999 synergistically reduces neuroblastoma cell viability, although medium doses show better synergy than high doses for arnebin due to strong effect from single therapy at high doses, and also considerably induces the expression of the Nrf2 target genes *HMOX1* and *NQO1*. Our data therefore suggest that JQ1 exerts synergistic anticancer effects with quinone-containing compounds, including the quinone-containing/DNA intercalator compounds, by blocking BRD3, BRD4 and Nrf2 binding to the Nrf2 target gene promoters, preventing Nrf2 target antioxidant gene transcription.

BRD4 plays an essential role in promoting mitotic progression by designating genes for timely activation at the end of mitosis and at the early G_1_ phase [31], and plays an essential role in inducing mitotic spindle assembly formation and preventing mitotic catastrophe [37]. Furthermore, inhibition or over-expression of BRD4 leads to cell cycle arrest at the G_2_/M and G_1_/S phases, respectively^38, 39^. On the other hand, anti-microtubule drugs arrest cells at mitosis, and the anti-microtubule drug nocodazole has been shown to induce rapid BRD4 protein release from chromatin [40, 41].

We have found that nine anti-microtubule drugs exerted strong synergy with JQ1, and that vincristine is the FDA-approved drug which exerts the best synergy with JQ1 in reducing neuroblastoma cell viability. Cell cycle and immunocytochemistry analyses show that JQ1 and vincristine synergistically induce the formation of aberrant mitotic spindles, and synergistically induce neuroblastoma cell cycle arrest at the G_2_/M phase and apoptosis. Our data suggest that neuroblastoma cells co-treated with JQ1 and anti-microtubule drugs are unable to recover from anti-microtubule drug-mediated cell cycle arrest at the mitotic phase due to JQ1-induced BRD4 inhibition.

Several BET bromodomain inhibitors, including GSK525762, OTX015, CPI-0610 and TEN-010, are currently in multiple clinical trials in patients with hematological malignancies or solid tumors including neuroblastoma. In this study, we have found that JQ1 treatment alone and vincristine treatment alone suppresses tumor growth in neuroblastoma-bearing mice, that JQ1 and vincristine co-operatively induce neuroblastoma cell growth inhibition and apoptosis *in vivo*, and that combination therapy with JQ1 and vincristine synergistically suppresses neuroblastoma progression in mice. As vincristine is the FDA-approved drug which exerts the best synergistic anticancer effects with JQ1 in our drug screen, this study provides direct evidence for the addition of vincristine, rather than other FDA-approved anticancer agents, in patients treated with BET bromodomain inhibitors in clinical trials.

## MATERIALS AND METHODS

### Cell culture

BE(2)-C neuroblastoma cells were cultured in Dulbecco's modified Eagle's medium supplemented with 10% fetal calf serum (FCS). Kelly and CHP134 neuroblastoma cells were cultured in RPMI-1640 medium supplemented with 10% FCS and 1% L-glutamine. WI38 fibroblast cells were cultured in minimum essential medium supplemented with 10% FCS. BE(2)-C and WI38 cells were obtained from American Type Culture Collection, and CHP134 and Kelly cells from European Collection of Cell Cultures. The identity of all cell lines was authenticated by small tandem repeat profiling conducted at the Garvan Institute or Cellbank Australia.

### Chemical compounds

JQ1 was obtained from Dr Jay Bradner at Dana-Farber Cancer Institute, Boston, MA, USA. A library of FDA-Approved Oncology Drugs, Natural Products, Diversity and Mechanistic sets consisting of 101, 120, 1597 and 879 compounds respectively, were provided by the National Cancer Institute, USA (http://dtp.nci.nih.gov/branches/dscb/repo_open.html). Additional Nanaomycin was purchased from ApexBio (ApexBio, Houston, TX).

### Alamar blue assays

Cells were incubated with Alamar blue, and read on a microplate reader at 570/595 nm. Results were calculated according to optical density absorbance units, and expressed as percentage change in the number of cells relative to controls [27, 42].

### Initial compound library screen for JQ1 enhancers

BE(2)-C neuroblastoma cells were seeded into 96-well plates at 3 × 10^3^ cells/well. Twenty-four hours later, the cells were treated in triplicate with control, JQ1 at 200nM, small molecule compounds at 10μM or 1μM, or JQ1 plus the compounds for 72 hours. Cell viability was determined by Alamar blue (Life Technologies, CA, USA) assays. Synergistic interaction between the compounds and JQ1 was calculated using fractional product method [22].

### Secondary screen for JQ1 enhancers

Shortlisted “hits” were subject to a more robust secondary screen at multiple concentrations in BE(2)-C neuroblastoma cells. Cells were seeded into a 96-well microtiter plate, incubated for 24 hours, and then treated with a range of doses of JQ1 (0, 125, 250, 500 and 1000nM), a range of doses of the compounds (0, 1, 2, 3.90625, 7.8125, 15.625, 31.25, 62.5, 125, 250, 500 and 1000nM), or combination of JQ1 and the compounds for 72 hours, followed by Alamar blue assays.

### Affymetrix microarray differential gene expression study

BE(2)-C neuroblastoma cells were treated with control, 500nM JQ1, 125nM nanaomycin, or combination for six hours, followed by RNA extraction. Differential gene expression was investigated using Affymetrix HuGene-1_0-st-v1 Arrays (Affymetrix, Santa Clara, CA), according to the manufacturer's instructions. The LimmaGP module (Gene Pattern, Broad Institute, Cambridge, MA) was employed to identify genes differentially expressed among samples using 1.8-fold change as the cut-off.

### siRNA transfection

Neuroblastoma cells were transfected with siRNAs from Qiagen using Lipofectamine 2000 (Invitrogen, Carlsbad, CA) reagent as previously described [27, 43].

### Apoptosis analysis

BE(2)-C, CHP134 and Kelly neuroblastoma and WI38 embryonic fibroblast cells were treated with control, JQ1, nanaomycin, combination of JQ1 and nanaomycin, vincristine, or combination of JQ1 and vincristine for 24 hours. The cells were then co-stained with FITC-conjugated Annexin-V (BD Biosciences, NJ, USA) and propidium iodide (BD Biosciences), followed by FACS analysis.

### Cell cycle analysis

Cells were treated with control, 500nM JQ1, 8nM vincristine, or combination of JQ1 and vincristine for 24 hours. Cells were then fixed in 80% ice-cold ethanol and stained with propidium iodide. The cells were finally subjected to cell cycle analysis with the FACS Canto Flow Cytometer and analyzed with FlowJo software.

### Immunocytochemistry

BE(2)-C, CHP134 and Kelly cells were seeded into Lab-Tak™ Chamber slides (Thermo Scientific, Pittsburgh, PA), and treated with vehicle control, 500nM, 8nM vincristine, or combination for 24 hours. Cells were then fixed for 15 minutes in 4% paraformaldehyde, blocked with 2% horse serum, and incubated with mouse anti-α-tubulin antibody (Sigma, Ct Louis, MO), Alexa Fluor 594-conjugated goat anti-mouse antibody (Thermo Scientific) and DAPI (Vector Laboratories, Burlingame, CA, USA). Cell images were captured under a fluorescence microscope (Zeiss, Oberkochen, Germany), and cells with aberrant mitotic spindles were quantified.

### Immunohistochemistry analysis

Mouse tumor tissues were sectioned, de-paraffinized and rehydrated. After antigen retrieval, tumor tissue sections were incubated with rabbit anti-Ki67 antibody (Abcam, Cambridge, MA) overnight, followed by incubation with biotinylated anti-rabbit antibody, streptavidin peroxidase and diaminobenzidine (Dako, Denmark). Tumor tissues were also subjected to TUNEL staining using the TUNEL kit (Roche, Basel, Switzerland), and visualized with diaminobenzidine. Tumor sections were counterstained with haematoxylin. Cells positively stained with the anti-Ki67 antibody or TUNEL were quantified as described previously [27].

### Chromatin immunoprecipitation (ChIP) assays

ChIP assays were performed with 2.5μg anti-Nrf2 (Santa Cruz, Dallas, Texas), anti-BRD3 (Bethyl Lab, Montgomery, Texas), anti-BRD4 (Bethyl Lab) or control antibody (Santa Cruz), followed by real-time PCR with primers targeting a negative control region or the promoter region of the *HMOX1* gene [44]. Fold enrichment of the antibody at the *HMOX1* gene promoter region was calculated by dividing PCR threshold from the anti-Nrf2, BRD3 or BRD4 antibody by PCR threshold from the control IgG, relative to the negative control region.

### Luciferase reporter assays

BE(2)-C neuroblastoma cells were transfected with a pGL3 construct expressing empty vector or the *HMOX1* gene promoter [44] for 24 hours, followed by treatment with vehicle control, 500nM JQ1, 1000nM nanaomycin, or combination for another 24 hours. Luciferase activity was measured, and expressed as fold change relative to the vehicle control treated samples transfected with the empty vector-pGL3 construct.

### Protein co-immunoprecipitation

HEK293 cells were co-transfected with pcDNA3.1-FLAG Nrf2 [44] construct, together with pJTI-R4-DEST-CMV-N-EmGFP empty vector (Life Technology, Carlsbad, CA), pJTI-R4-DEST-CMV-N-EmGFP BRD3 (Addgene, Cambridge, MA), pcDNA4-HA empty vector or pcDNA4-To-HA-BRD4FL (Addgene) construct, using Lipofectamine LTX (Life Technology) for 48 hours, followed by protein extraction. Protein extracts from 1500μg of cell lysate were immunoprecipitated with 5μg of anti-BRD3 or anti-BRD4 antibody (Bethyl Laboratories) overnight at 4°C. Immunoprecipitates were captured by Protein G-Sepharose (GE Healthcare, Buckinghamshire, England), and then released by boiling in sample buffer, followed by immunoblot analysis with anti-BRD3, anti-BRD4 and anti-Nrf2 (Merck Millipore, Billerica, MA) antibodies.

### Experimental therapy in mice

The animal experiments were approved by the Animal Care and Ethics Committee of the local institute. Five-to-six week old nude mice were injected subcutaneously with 1 × 10^7^ Kelly cells into the flank. When tumors reached 0.05cm^3^, the mice were treated intraperitoneally with control solvent, JQ1 (50 mg/kg daily), vincristine (0.2 mg/kg every second day), or combination of JQ1 (50 mg/kg daily) and vincristine (0.2 mg/kg every second day) for three weeks. Tumor development was monitored by measuring the tumors with a caliper [tumor volume = (length × width × height)/2]. Mice were culled at the end of the treatments, and tumors were collected, formalin-fixed and paraffin-embedded.

### Statistical analysis

For statistical significant difference analysis, unpaired t-test for two groups or ANOVA among groups was used. All statistical tests were two-sided. For synergy analysis of the combination therapy in the initial and secondary drug screens, combination effects were examined with the fractional product method [22]. For further cell viability assays in which multiple doses of JQ1 and compounds were used, synergy was determined by the median-drug effect analysis method developed by Chou and Talalay [45]. A combination index (CI) was calculated using the Calcusyn software, where CI < 1, 1, or > 1 indicates synergistic, additive and antagonistic effect respectively.

## SUPPLEMENTARY MATERIALS








