# Supplementary material for: The BET bromodomain inhibitor exerts the most potent synergistic anticancer effects with quinone-containing compounds and anti-microtubule drugs
Source: Oncotarget. 2016 Oct 13;7(48):79217–32. doi: 10.18632/oncotarget.12640 (PMC5346709; doi:10.18632/oncotarget.12640)
Supplement: Supplementary file 1 [file oncotarget-07-79217-s001.pdf]

The BET bromodomain inhibitor exerts the most potent synergistic anticancer effects with quinone-containing compounds and anti-microtubule drugs

SUPPLEMENTARY MATERIALS

Primers for ChIP assays

The sequences of specific primers used for ChIP assays were: 5'-GAAGAGTCCCCAGCTGAACA-3' (forward) and 5'-GACCTCTTCACCCTGCAAAA-3' (reverse) for negative control region (-20000bp upstream of the HMOX1 gene transcription start site); and 5'-CCAGAAAGTGGGCATCAGCT-3' (forward) and 5'-GTCACATTTATGCTCGGCGG-3' (reverse) for the HMOX1 gene promoter region.

Primers for real-time RT-PCR

The sequences of specific real-time RT-PCR primers were: 5'-TTTGGCCACCTTCAATAAGC-3' (forward) and 5'-GGTCCTCCAATGAGTCTCCA-3' (reverse) for DNMT3B; 5'-AACTTTCAGAAGGGCCAGGT-3' (forward) and 5'-GTAGACAGGGGCGAAGACTG-3' (reverse) for HMOX1; 5'-CAGCTCACCGAGAGCCTAGT-3' (forward) and 5'-GCCTCCTTCATGGCATAGTT-3' (reverse) for NQO1; and 5'-AGCCCAGCACATCCAGTCA-3' (forward) and 5'-TGTGGGCAACCTGGGAGTAG-3' (reverse) for Nrf2; and 5'-AGGCCAACCGCGAGAAG-3' and 5'-ACAGCCTGGATAGCAACGTACA-3' for β-actin.

siRNAs

Predesigned siRNAs targeting DNMT3B (Cat. No. SI00092967 and SI04987157) and Nrf2 (SI03246614) were purchased from Qiagen (Valencia, CA, USA).

A

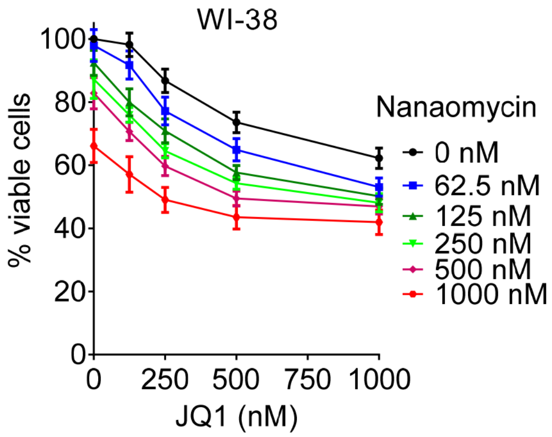

B

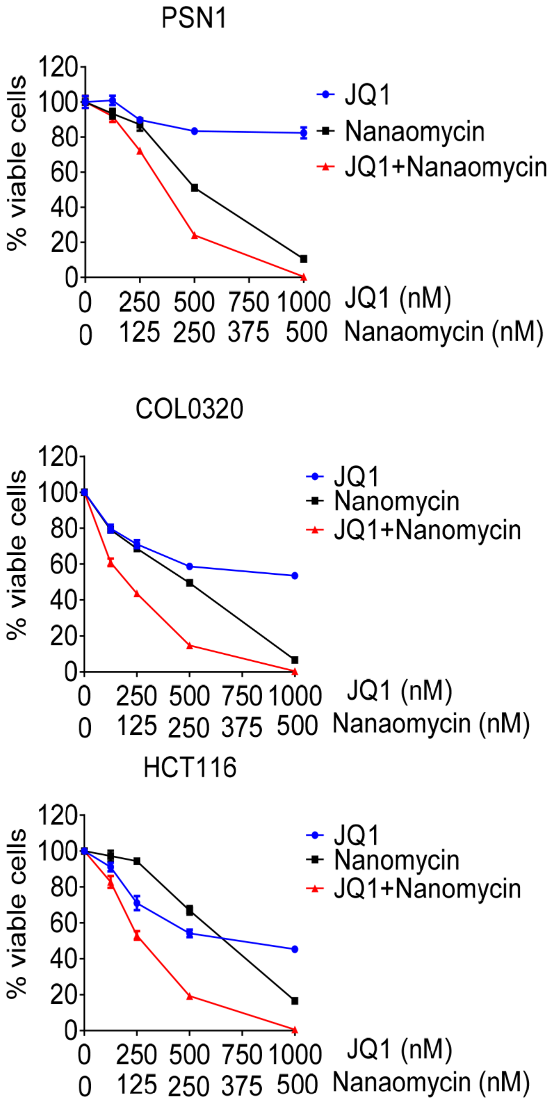

C

	CI at inhibition of		
Cell line	75%	90%	r[a]
PSN1	0.79	0.69	0.97
COL0320	0.51	0.39	0.94
HCT116	0.56	0.45	0.97

Supplementary Figure S1: The quinone-containing compound nanaomycin and the BET bromodomain inhibitor JQ1 do not exert considerable cytotoxicity against normal embryonic fibroblasts, but synergistically reduce the number of c-Myc over-expressing cancer cells. A-C. WI38 embryonic fibroblasts, c-Myc over-expressing PSN1 pancreatic, COLO320 and HCT116 colon cancer cells were treated with vehicle control, JQ1, nanaomycin, or JQ1 plus nanaomycin at the indicated doses for 72 hours, followed by Alamar Blue assays. Relative numbers of cells were expressed as a percentage compared with samples treated with vehicle control. All experiments were repeated for at least three times. Error bars represent standard error. C. CI values were determined for a mutually exclusive interaction using the Calcusyn program; a CI of <1, 1, or >1 indicates synergistic, additive and antagonistic effects respectively. CI_{75} and CI_{90} represented CIs for 75 and 90% reduction in the number of viable cells respectively. r^a is the linear correlation coefficient of the median-effect plot.

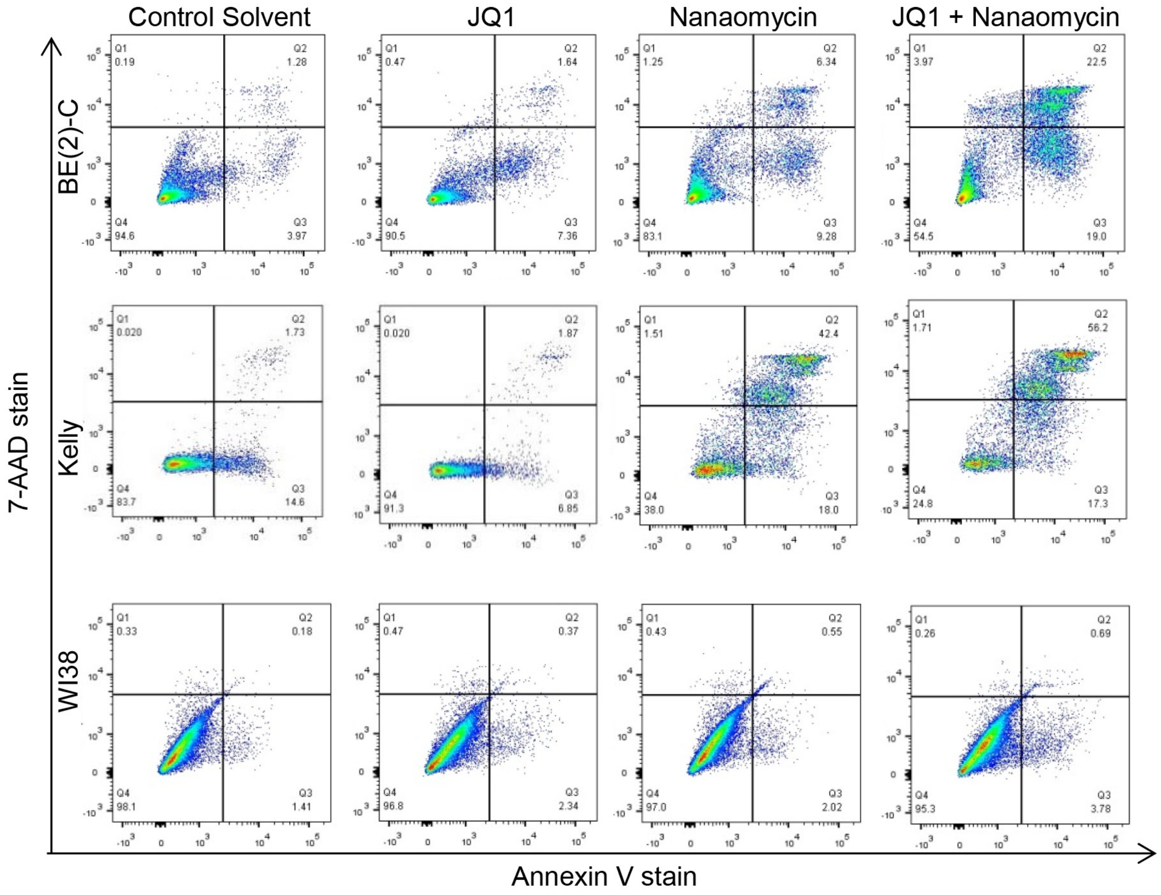

Supplementary Figure S2: JQ1 and nanaomycin synergistically induce apoptosis in neuroblastoma but not normal fibroblast cells. BE(2)-C, Kelly neuroblastoma and WI38 fibroblast cells were treated with vehicle control, 500nM JQ1 and/or 1000nM nanaomycin for 72 hours, followed by Annexin V and 7-AAD staining and flow cytometry analysis of positively stained cells. Q1 consisted of necrotic cell population (7-AAD positive), Q2 late apoptotic cell population (Annexin V & 7-AAD positive), Q3 early apoptotic cell population (Annexin V positive) and Q4 live cell population (unstained). The graph showed one representative of three independent replicate experiments.

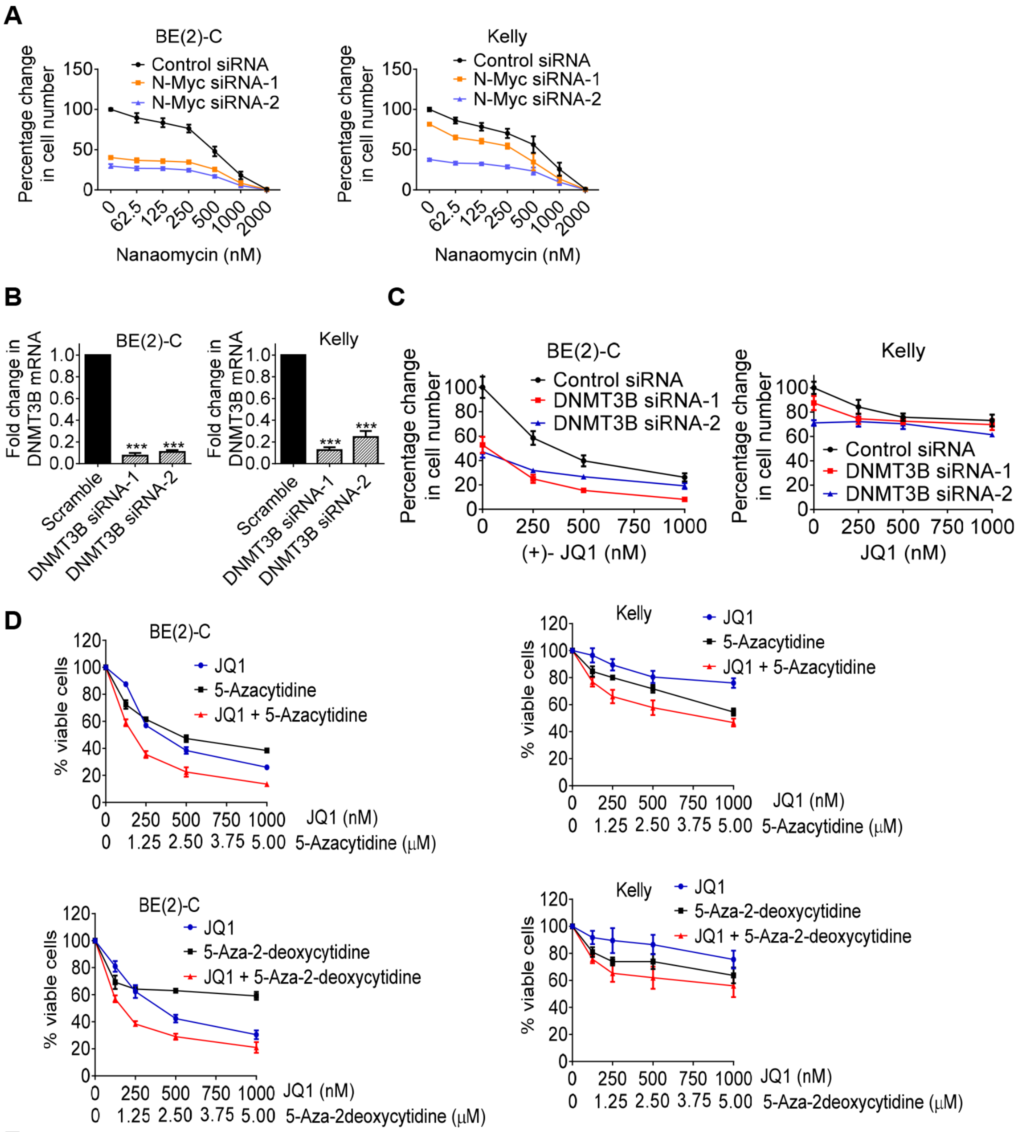

E

Cell line	Combination JQ1 +	CI at inhibition of 75%	CI at inhibition of 90%	r[a]
BE(2)-C	Azacytidine	0.69	0.73	0.99
Kelly	Azacytidine	1.34	2.20	1.00

Cell line	Combination JQ1 +	CI at inhibition of 75%	CI at inhibition of 90%	r[a]
BE(2)-C	5'-Aza-2-deoxycytidine	0.60	0.94	0.99
Kelly	5'-Aza-2-deoxycytidine	0.72	1.29	0.97

Supplementary Figure S3: Suppression of N-Myc does not synergize with nanaomycin, and suppression of DNMT3B does not synergize with JQ1, to exert anticancer effects. A. BE(2)-C and Kelly neuroblastoma cells were transfected with control siRNA, N-Myc siRNA-1 or N-Myc siRNA-2. Twenty-four hours later, cells were treated with vehicle control or a range of doses of nanaomycin for 72 hours. The numbers of viable cells were examined by Alamar blue assays. **B.** BE(2)-C and Kelly cells were transfected with control siRNA, DNMT3B siRNA-1 or DNMT3B siRNA-2, followed by RT-PCR analysis of DNMT3B mRNA expression 48 hours after siRNA transfection. **C.** BE(2)-C and Kelly cells were transfected with control siRNA, DNMT3B siRNA-1 or DNMT3B siRNA-2 for 24 hours, then treated with control or JQ1 at the indicated doses for another 72 hour followed by Alamar blue assays. **D-E.** BE(2)-C and Kelly cells were treated with a range of doses of JQ1 and/or a range of doses of the DNMT inhibitor 5-aza-2-deoxycytidine or 5-azacytidine for 72 hours. The numbers of viable cells were examined by Alamar blue assays 72 hours later. E. CI values were determined for a mutually exclusive interaction using the Calcusyn program; a CI of <1, 1, or >1 indicates synergistic, additive and antagonistic effects respectively. CI_{75} and CI_{90} represented CIs for 75 and 90% reduction in the number of viable cells respectively. r[a] is the linear correlation coefficient of the median-effect plot.

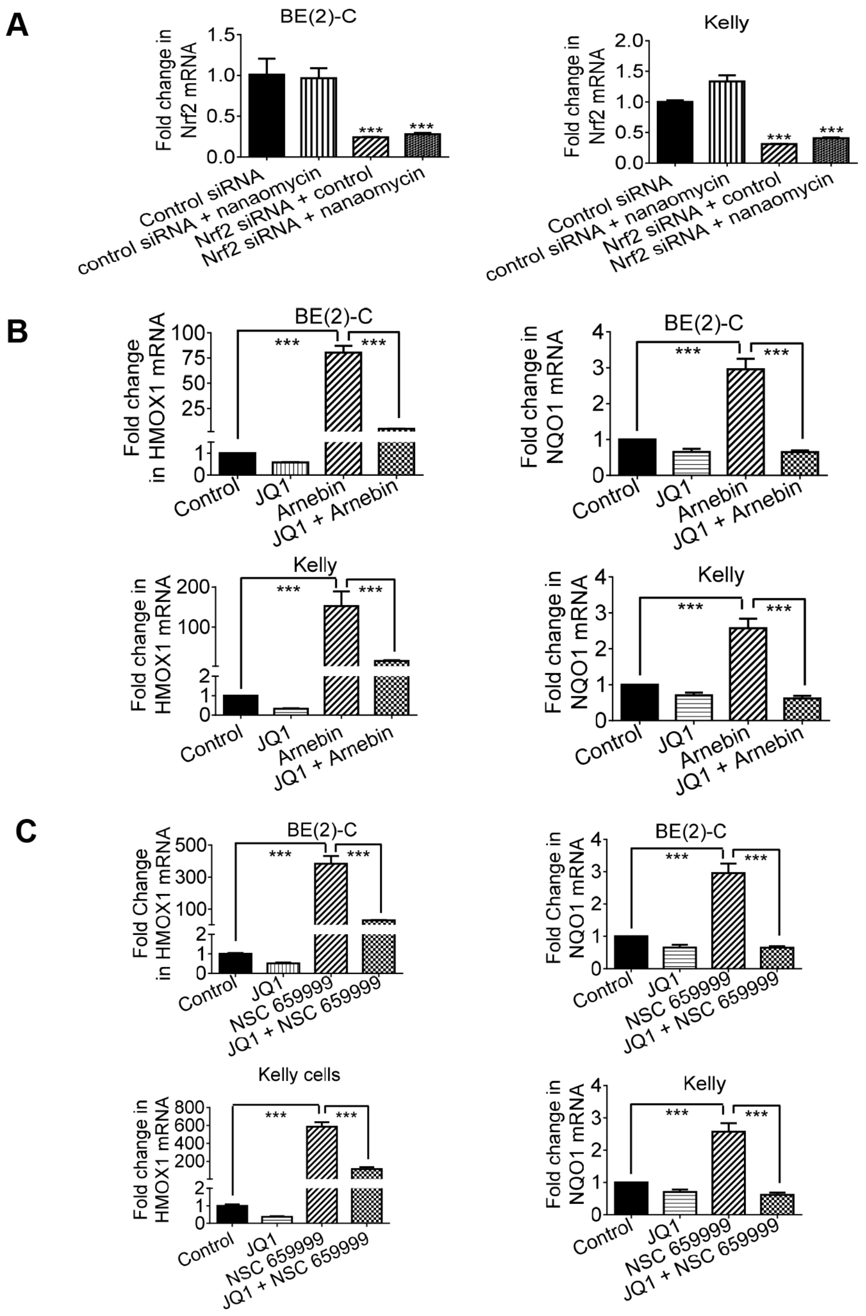

Supplementary Figure S4: The quinone-containing compounds nanaomycin and arnebin and the quinone-containing/ DNA intercalator compound NSC659999 activate Nrf2 target gene expression. A. BE(2)-C and Kelly neuroblastoma cells were transfected with control siRNA or Nrf2 siRNA and treated with 1000nM nanaomycin for 48 hours, followed by RT-PCR analysis of Nrf2 expression. **B-C.** BE(2)-C and Kelly cells were treated with vehicle control, 500nM JQ1, 125nM arnebin, 500nM JQ1 plus 125nM arnebin (B), 1000nM compound NSC659999, or 500nM JQ1 plus 1000nM compound NSC659999 (C) for 48 hours, followed by RT-PCR analysis of HMOX1 and NQO1 expression. Error bar represented standard error. ***$P < 0.001$.

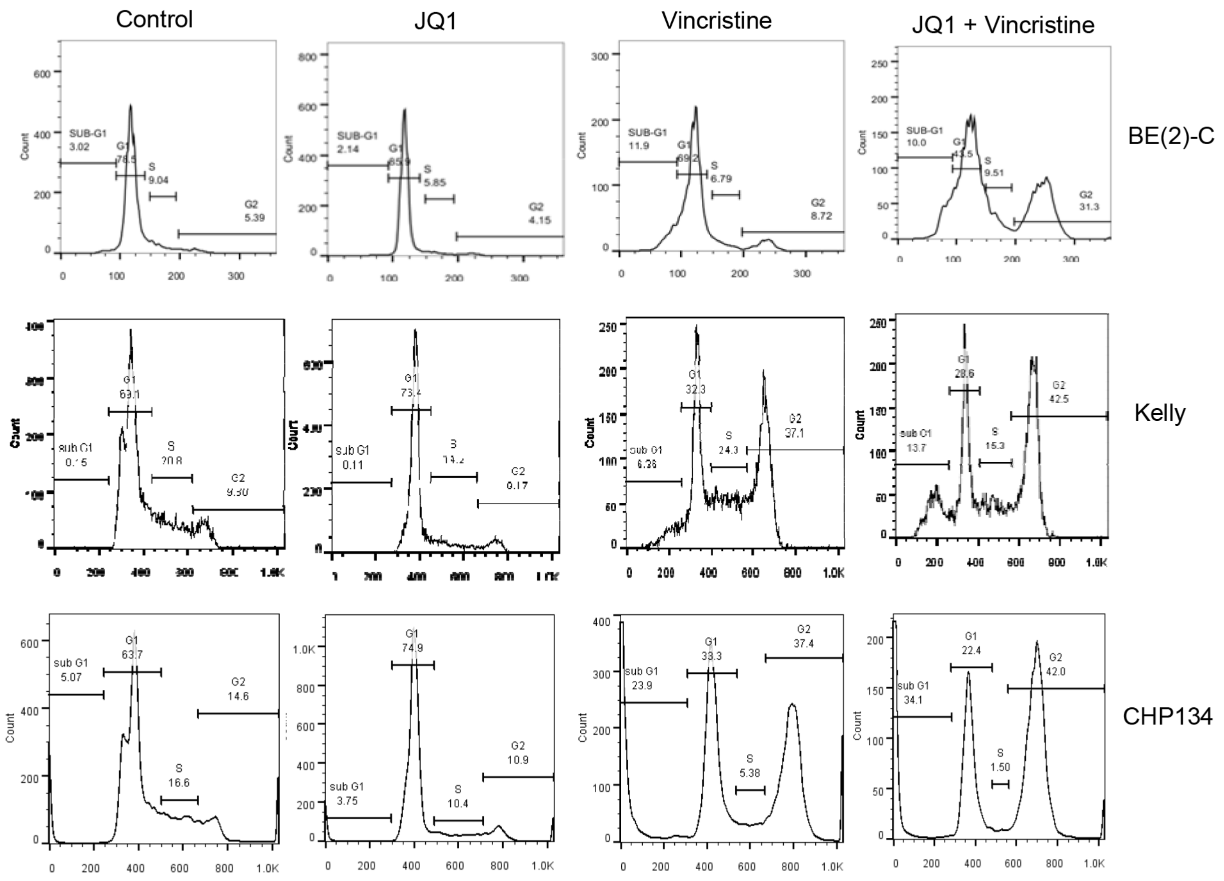

Supplementary Figure S5: JQ1 and vincristine synergistically induce neuroblastoma cell cycle arrest at the G_2/M phase and cell death. BE(2)-C, Kelly and CHP134 neuroblastoma cells were treated with vehicle control, 500nM JQ1 and/or 8nM vincristine for 24 hours, followed by propidium iodide staining. The cells were then examined with a FACS Canto Flow Cytometer, and cell cycle was analyzed with FlowJo software. The graph showed one representative of three independent replicate experiments.

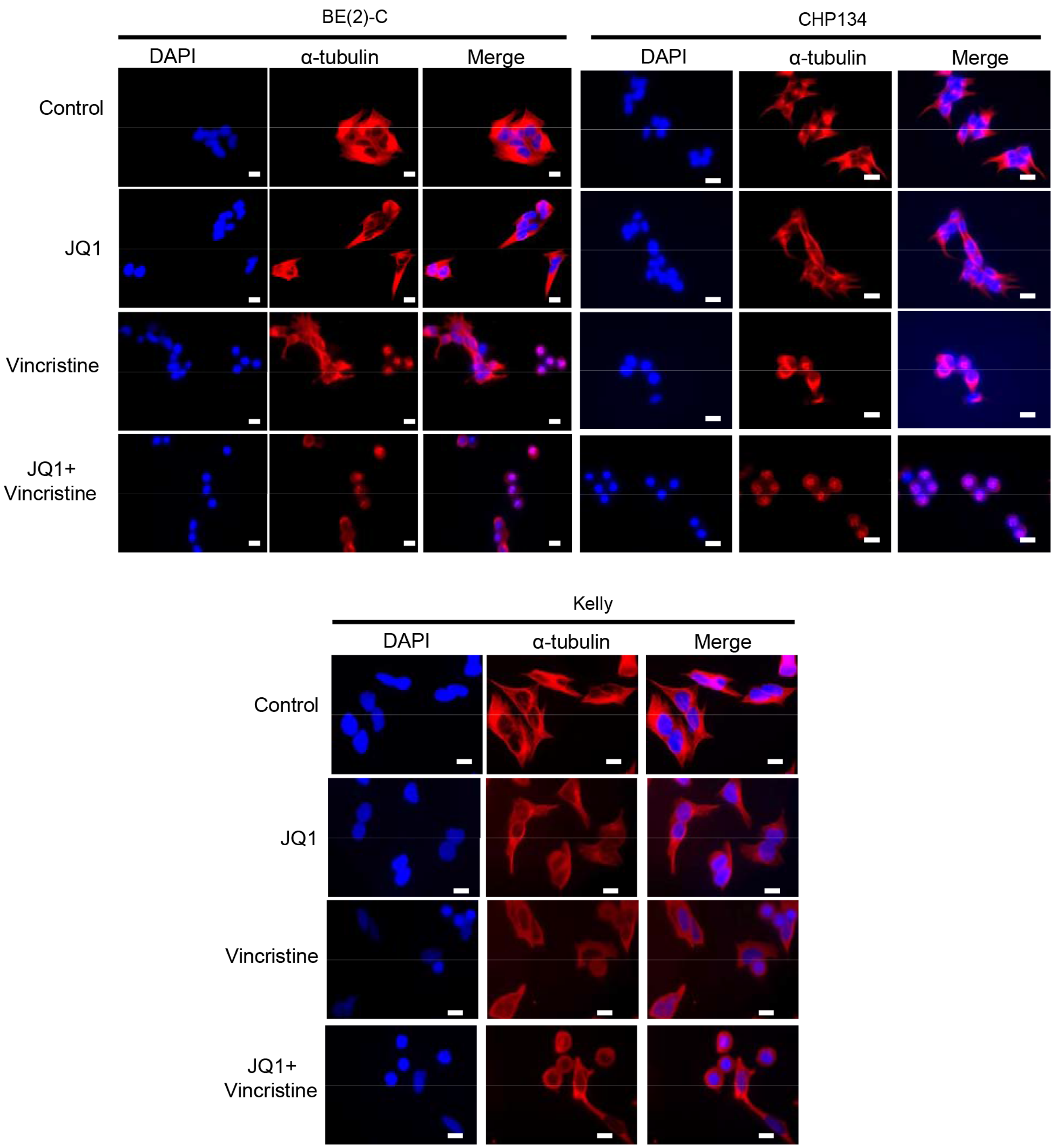

Supplementary Figure S6: JQ1 and vincristine synergistically induce aberrant mitotic spindle formation in neuroblastoma cells. BE(2)-C, Kelly and CHP134 neuroblastoma cells were treated with vehicle control, 500nM JQ1 and/or 8nM vincristine for 24 hours followed by immunofluorescence staining with an anti-α-tubulin antibody. DNA was counterstained with DAPI. Scale bars represent 10µm. The graph showed one representative of three independent replicate experiments.

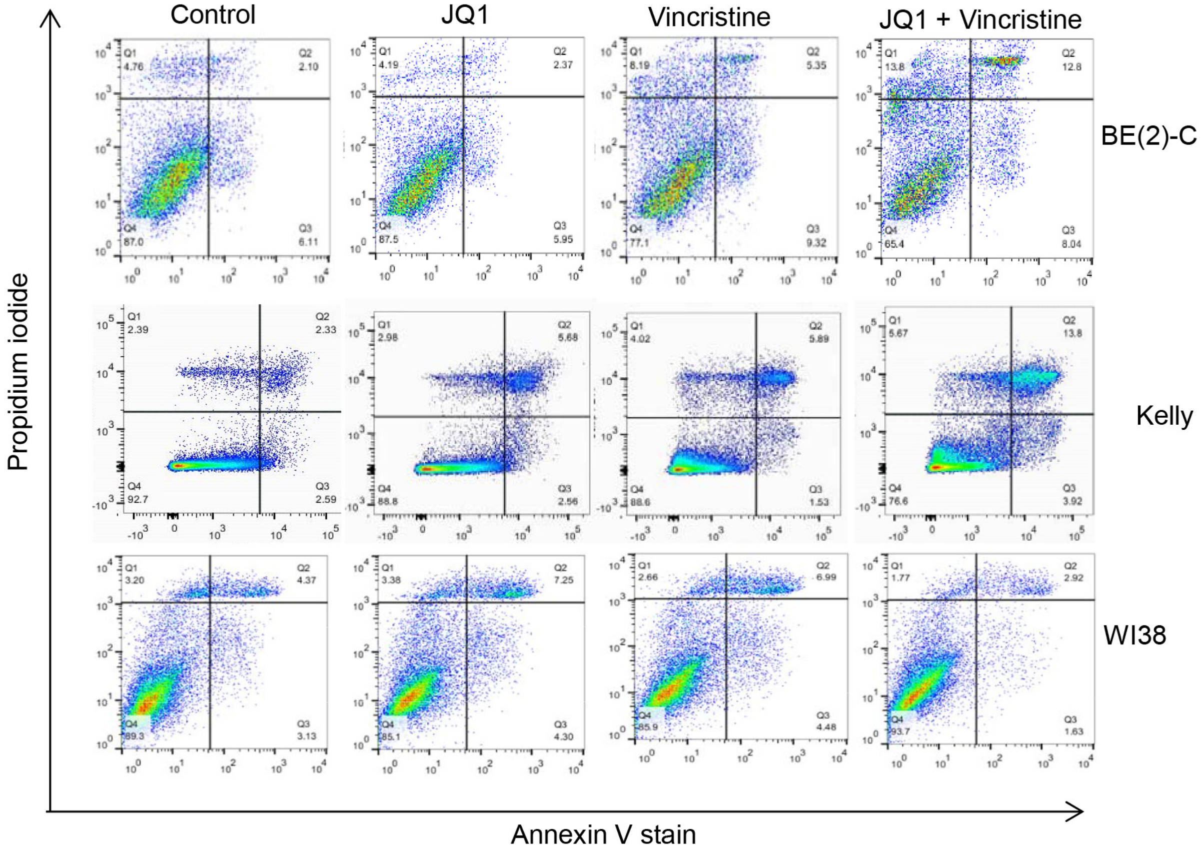

Supplementary Figure S7: JQ1 and vincristine synergistically induce apoptosis in neuroblastoma but not normal embryonic fibroblast cells. BE(2)-C, Kelly and WI38 cells were treated with vehicle control, 500nM JQ1 (1000nM for Kelly and WI38 cells), 8nM vincristine (16nM for WI38), or JQ1 plus vincristine for 24 hours, followed by Annexin V and propidium iodide staining and flow cytometry analysis of cells positively stained by Annexin V and/or propidium iodide. Q1 consisted of necrotic cell population (positively stained by propidium iodide), Q2 late apoptotic cell population (positively stained by Annexin V & propidium iodide), Q3 early apoptotic cell population (positively stained by Annexin V) and Q4 live cell population (negatively stained by Annexin V and propidium iodide). The graph showed one representative of three independent replicate experiments.

Supplementary Table S1: Nrf2 target genes up-regulated by nanaomycin were negatively regulated by JQ1

Probe ID	Gene Symbol	Fold change by Nanaomycin	Fold change by JQ1	Fold change by Nanaomcin + JQ1
**8072678**	HMOX1	14.61	-1.70	6.67
**8118509**	PPT2	4.34	1.48	1.93
**7917779**	GCLM	3.44	-1.09	3.07
**7931832**	AKR1C2	2.24	-0.60	-1.18
**8180376**	AKR1C1	1.96	1.31	1.41
**8002303**	NQO1	1.92	-1.17	1.21

BE(2)-C neuroblastoma cells were treated with vehicle control, JQ1, nanaomycin or combination of JQ1 and nanaomycin for 6 hours, followed by Affymetrix microarray analysis of differential gene expression. Six genes regulated by nanaomycin by more than 1.8 fold were negatively regulated by JQ1.

Supplementary Dataset S1: Compounds which reduced the numbers of viable BE(2)-C cells by ≥ 90% on their own as well as in combination with JQ1, as compared with vehicle control-treated samples, in the initial compound library screening.

See Supplementary File 1

Supplementary Dataset S2: List of compounds which exerted synergistic anticancer effects with JQ1 with combination R values between 0.4 and 0.7 in the secondary screen.

See Supplementary File 2

Supplementary Dataset S3: List of compounds which exerted strong synergistic anticancer effects with JQ1 with combination R values less than 0.4 in the secondary screen.

See Supplementary File 3
