# Supplementary material for: The BET bromodomain inhibitor exerts the most potent synergistic anticancer effects with quinone-containing compounds and anti-microtubule drugs
Source: Oncotarget. 2016 Oct 13;7(48):79217–32. doi: 10.18632/oncotarget.12640 (PMC5346709; doi:10.18632/oncotarget.12640)
Supplement: Supplementary file 2 [file oncotarget-07-79217-s002.docx]

**Supplementary Dataset S1.** Compounds which reduced the numbers of viable BE(2)-C cells by ≥ 90% on their own as well as in combination with JQ1, as compared with vehicle control-treated samples, in the initial compound library screening.

| **Plate ID** | **Well ID** | **NSC number** | **% viable cells (compound alone)** | **% viable cells (compound + JQ1)** |
| --- | --- | --- | --- | --- |
| 4751 | D10 | 219734 | 1 | 10 |
| 4751 | G08 | 269146 | 8 | 10 |
| 4743 | G05 | 1906 | 6 | 10 |
| 4723 | B10 | 30205 | 2 | 10 |
| 4744 | C05 | 605756 | 9 | 10 |
| 13120880 | B05 | 23969 | 6 | 10 |
| 13120880 | B07 | 36351 | 10 | 9 |
| 4732 | E02 | 299514 | 9 | 9 |
| 13120880 | D09 | 76022 | 2 | 9 |
| 4744 | F03 | 175493 | 10 | 9 |
| 4762 | E09 | 609699 | 8 | 9 |
| 4743 | A07 | 65423 | 9 | 8 |
| 13120881 | E05 | 270914 | 7 | 8 |
| 4733 | B03 | 228150 | 1 | 8 |
| 4751 | E10 | 243928 | 9 | 8 |
| 4743 | D11 | 667251 | 6 | 8 |
| 4723 | C05 | 641396 | 1 | 8 |
| 4748 | A09 | 211489 | 10 | 8 |
| 4734 | A06 | 143241 | 8 | 8 |
| 4751 | H04 | 328426 | 3 | 7 |
| 4745 | C10 | 69187 | 8 | 7 |
| 4738 | C10 | 57103 | 1 | 7 |
| 4734 | A11 | 143491 | 3 | 7 |
| 4728 | B06 | 639174 | 6 | 7 |
| 4720 | G02 | 21683 | 7 | 7 |
| 4727 | B05 | 117028 | 9 | 7 |
| 4747 | D06 | 629971 | 8 | 7 |
| 4763 | B02 | 761432 | 9 | 7 |
| 4745 | H11 | 651079 | 7 | 7 |
| 4750 | H09 | 671394 | 1 | 7 |
| 4732 | B11 | 135168 | 2 | 7 |
| 4762 | A10 | 747971 | 6 | 7 |
| 4726 | B11 | 56410 | 6 | 7 |
| 4749 | F07 | 34391 | 5 | 7 |
| 4726 | B05 | 281383 | 8 | 6 |
| 4749 | F06 | 689857 | 5 | 6 |
| 4733 | A08 | 622691 | 9 | 6 |
| 4726 | A04 | 308848 | 2 | 6 |
| **Plate ID** | **Well ID** | **NSC number** | **% viable cells (compound alone)** | **% viable cells (compound + JQ1)** |
| 4733 | A06 | 339316 | 8 | 6 |
| 4732 | D06 | 33570 | 1 | 6 |
| 4725 | D03 | 275971 | 9 | 6 |
| 13120881 | D10 | 407286 | 6 | 6 |
| 4725 | E04 | 60785 | 10 | 6 |
| 13120880 | D06 | 32192 | 2 | 6 |
| 4751 | F10 | 700582 | 8 | 6 |
| 13120880 | F11 | 122750 | 6 | 5 |
| 4734 | A04 | 665497 | 5 | 5 |
| 4720 | F09 | 13151 | 5 | 5 |
| 4744 | A10 | 349155 | 5 | 5 |
| 4745 | C02 | 662553 | 10 | 5 |
| 4727 | A10 | 201631 | 9 | 5 |
| 13120880 | G06 | 35611 | 4 | 5 |
| 4746 | C07 | 44690 | 3 | 5 |
| 4727 | G07 | 670283 | 9 | 5 |
| 4731 | C11 | 168225 | 7 | 5 |
| 4762 | E08 | 757441 | 9 | 5 |
| 4749 | D08 | 96932 | 3 | 5 |
| 4746 | C09 | 376265 | 10 | 5 |
| 4745 | E07 | 667467 | 7 | 5 |
| 4731 | F07 | 92937 | 1 | 5 |
| 13120881 | F04 | 250429 | 2 | 5 |
| 4731 | D03 | 30813 | 7 | 5 |
| 4732 | C06 | 26980 | 1 | 4 |
| 13120881 | C05 | 263164 | 4 | 4 |
| 4733 | D05 | 328010 | 8 | 4 |
| 4744 | E04 | 269142 | 3 | 4 |
| 4750 | E09 | 659999 | 2 | 4 |
| 4762 | D10 | 760766 | 1 | 4 |
| 4744 | G09 | 267033 | 4 | 4 |
| 4734 | E02 | 325014 | 4 | 4 |
| 4722 | H11 | 158383 | 3 | 4 |
| 4750 | A10 | 679524 | 3 | 4 |
| 4728 | B08 | 122819 | 1 | 4 |
| 4731 | A11 | 195327 | 9 | 4 |
| 4748 | E03 | 687849 | 2 | 4 |
| 4731 | A10 | 242557 | 9 | 4 |
| 4732 | B06 | 22070 | 1 | 4 |
| 13120881 | D11 | 719655 | 4 | 3 |
| 4747 | C09 | 172946 | 3 | 3 |
| 4749 | E08 | 102815 | 3 | 3 |
| 4752 | D03 | 333856 | 8 | 3 |
|  |  |  |  |  |
| **Plate ID** | **Well ID** | **NSC number** | **% viable cells (compound alone)** | **% viable cells (compound + JQ1)** |
| 4762 | A11 | 123127 | 4 | 3 |
| 4744 | A03 | 145669 | 2 | 3 |
| 13120880 | F07 | 45923 | 4 | 3 |
| 4763 | C02 | 125973 | 5 | 3 |
| 4749 | D06 | 673622 | 3 | 3 |
| 4750 | F04 | 268251 | 2 | 3 |
| 13120881 | D08 | 338250 | 1 | 3 |
| 4750 | B11 | 7525 | 4 | 3 |
| 4762 | H06 | 26980 | 2 | 3 |
| 4727 | F09 | 136513 | 2 | 3 |
| 4750 | A03 | 172924 | 1 | 2 |
| 4749 | E03 | 639828 | 2 | 2 |
| 4724 | D05 | 94600 | 3 | 2 |
| 4752 | G02 | 18268 | 2 | 2 |
| 4752 | F02 | 3053 | 2 | 2 |
| 4751 | B03 | 136044 | 2 | 2 |
| 4750 | A11 | 4320 | 3 | 2 |
| 13120880 | E10 | 96911 | 1 | 2 |
| 4750 | G11 | 52141 | 4 | 2 |
| 4745 | H08 | 106408 | 2 | 2 |
| 4721 | F10 | 93427 | 3 | 2 |
| 4762 | G11 | 122819 | 2 | 2 |
| 4744 | D03 | 165897 | 2 | 2 |
| 4762 | H11 | 246131 | 3 | 2 |
| 4762 | F08 | 279836 | 2 | 2 |
| 4751 | C07 | 7532 | 4 | 2 |
| 4737 | A05 | 71795 | 3 | 2 |
| 4750 | B02 | 132791 | 2 | 2 |
| 4734 | F10 | 654260 | 2 | 2 |
| 4749 | A07 | 7521 | 4 | 2 |
| 4762 | F10 | 747973 | 4 | 2 |
| 4744 | F08 | 63701 | 2 | 2 |
| 4727 | C07 | 607097 | 2 | 2 |
| 4746 | E05 | 632841 | 3 | 2 |
| 4734 | A10 | 343256 | 1 | 2 |
| 4763 | H02 | 3053 | 2 | 2 |
| 4751 | B09 | 325319 | 2 | 2 |
| 4750 | H11 | 65104 | 1 | 2 |
| 4746 | G05 | 635121 | 8 | 2 |
| 4751 | D09 | 526417 | 2 | 2 |
| 4751 | G03 | 208734 | 2 | 2 |
| 13120881 | E02 | 145118 | 9 | 2 |
|  |  |  |  |  |
| **Plate ID** | **Well ID** | **NSC number** | **% viable cells (compound alone)** | **% viable cells (compound + JQ1)** |
| 4751 | G07 | 58514 | 2 | 2 |
| 4751 | H09 | 243023 | 2 | 2 |
| 4762 | H10 | 82151 | 2 | 2 |
| 4750 | D04 | 258812 | 2 | 2 |
| 4750 | E04 | 267229 | 2 | 2 |
| 13120881 | D02 | 133100 | 2 | 2 |
| 4751 | H07 | 67574 | 1 | 2 |
| 4724 | B11 | 37168 | 4 | 2 |
| 13120880 | C02 | 2952 | 2 | 2 |
| 4751 | B02 | 70845 | 2 | 2 |
| 4745 | E04 | 175636 | 2 | 2 |
| 13120880 | D10 | 94600 | 2 | 2 |
| 4747 | E08 | 126727 | 1 | 2 |
| 4763 | A03 | 24559 | 3 | 2 |
| 4750 | D06 | 354844 | 2 | 2 |
| 13120881 | F08 | 345647 | 2 | 2 |
| 4745 | B04 | 147340 | 1 | 2 |
| 4733 | A07 | 352890 | 3 | 2 |
| 4745 | A05 | 266535 | 2 | 2 |
| 4728 | D06 | 45383 | 1 | 2 |
| 13120880 | C11 | 118343 | 1 | 2 |
| 4750 | H04 | 269754 | 1 | 2 |
| 4750 | G05 | 328166 | 1 | 2 |
| 13120880 | G05 | 30552 | 2 | 2 |
| 13120880 | C06 | 31867 | 1 | 2 |
| 4736 | C03 | 2805 | 4 | 2 |
| 4750 | F02 | 165563 | 3 | 2 |
| 4748 | F10 | 349644 | 2 | 2 |
| 4732 | B10 | 96541 | 3 | 2 |
| 13120880 | B09 | 71795 | 1 | 1 |
| 4751 | H08 | 301460 | 2 | 1 |
| 13120880 | E11 | 122224 | 1 | 1 |
| 13120881 | E07 | 330753 | 1 | 1 |
| 4747 | A08 | 24817 | 2 | 1 |
| 13120880 | D07 | 42038 | 1 | 1 |
| 13120880 | D11 | 122023 | 1 | 1 |
| 4733 | C06 | 339594 | 1 | 1 |
| 13120880 | F08 | 63701 | 3 | 1 |
| 13120881 | E09 | 375294 | 2 | 1 |
| 4746 | B09 | 323241 | 1 | 1 |
| 4747 | C10 | 403148 | 1 | 1 |
| 4749 | G08 | 126728 | 1 | 1 |
|  |  |  |  |  |
| **Plate ID** | **Well ID** | **NSC number** | **% viable cells (compound alone)** | **% viable cells (compound + JQ1)** |
| 4746 | B06 | 637578 | 4 | 1 |
| 4732 | B03 | 311165 | 4 | 1 |
| 4733 | B08 | 637578 | 4 | 1 |
| 4726 | C04 | 303612 | 1 | 1 |
| 13120880 | B06 | 31048 | 1 | 1 |
| 4745 | G05 | 352890 | 1 | 1 |
| 13120881 | G08 | 349438 | 1 | 1 |
| 4751 | D07 | 24559 | 2 | 1 |
| 13120881 | G10 | 614552 | 1 | 1 |
| 4722 | D08 | 139021 | 3 | 1 |
| 4727 | D07 | 622689 | 1 | 1 |
| 4734 | G10 | 679525 | 1 | 1 |
| 4722 | G04 | 207895 | 1 | 1 |
| 4722 | B11 | 228155 | 1 | 1 |
| 4725 | C10 | 622608 | 1 | 1 |
| 13120880 | B03 | 7524 | 1 | 1 |
| 4750 | A02 | 129414 | 1 | 1 |
| 4750 | E07 | 638646 | 1 | 1 |
| 13120881 | G09 | 382796 | 1 | 1 |
| 4745 | E05 | 330770 | 1 | 1 |
| 13120880 | F10 | 105388 | 1 | 1 |
| 4726 | E04 | 300289 | 1 | 1 |
| 4721 | D11 | 98938 | 1 | 1 |
| 4734 | C11 | 268251 | 1 | 1 |
| 4733 | H06 | 349156 | 1 | 1 |
| 4746 | A08 | 143648 | 1 | 1 |
| 4728 | A06 | 322661 | 1 | 1 |
| 4745 | A03 | 65346 | 1 | 1 |
| 4749 | H07 | 45383 | 1 | 1 |
| 4733 | D08 | 7419 | 1 | 1 |
| 4752 | A04 | 49451 | 2 | 1 |
| 13120880 | C09 | 72116 | 1 | 1 |
| 13120881 | B02 | 129536 | 1 | 1 |
| 4734 | E10 | 354844 | 1 | 1 |
| 4752 | E04 | 613009 | 1 | 1 |
| 4724 | G06 | 105827 | 1 | 1 |
| 4763 | D02 | 49842 | 1 | 1 |
| 4734 | D11 | 330500 | 1 | 1 |
| 4751 | C04 | 265450 | 2 | 1 |
| 13120880 | C07 | 36398 | 1 | 1 |
| 4734 | F08 | 70931 | 1 | 1 |
| 4733 | F05 | 330770 | 1 | 1 |
|  |  |  |  |  |
| **Plate ID** | **Well ID** | **NSC number** | **% viable cells (compound alone)** | **% viable cells (compound + JQ1)** |
| 13120880 | C10 | 89671 | 1 | 1 |
| 4763 | C03 | 608210 | 1 | 1 |
| 4725 | D04 | 630602 | 1 | 1 |
| 4763 | E02 | 67574 | 1 | 1 |
| 13120880 | D03 | 8519 | 1 | 1 |
| 4751 | D03 | 153858 | 1 | 1 |
| 4744 | C02 | 89671 | 1 | 1 |
| 13120880 | B08 | 56464 | 1 | 1 |
| 4745 | B05 | 267461 | 1 | 1 |
| 4762 | B09 | 756645 | 1 | 1 |
| 13120880 | B11 | 114344 | 1 | 1 |
| 4762 | A08 | 681239 | 1 | 1 |
| 4762 | G10 | 754143 | 1 | 1 |
| 4748 | G04 | 24819 | 1 | 1 |
| 4762 | B06 | 701852 | 1 | 1 |
| 4723 | F09 | 26113 | 8 | 1 |
| 4724 | A07 | 111118 | 1 | 1 |
| 4734 | C02 | 309401 | 7 | 1 |
| 13120880 | C04 | 13252 | 1 | 1 |
| 4751 | G06 | 669356 | 1 | 1 |
| 4724 | A11 | 36758 | 1 | 1 |
| 4727 | E10 | 305798 | 1 | 1 |
| 13120880 | D08 | 60387 | 1 | 1 |
| 4748 | F04 | 24818 | 1 | 1 |
| 4725 | G11 | 13316 | 1 | 1 |
| 4748 | H04 | 33410 | 1 | 1 |
| 4727 | B09 | 65238 | 1 | 1 |
| 4742 | B11 | 1771 | 1 | 1 |
| 4763 | D03 | 758252 | 1 | 1 |
| 4750 | F10 | 690634 | 1 | 1 |
| 4739 | D03 | 150954 | 7 | 1 |
| 13120881 | D09 | 369397 | 1 | 1 |
| 4762 | D09 | 14229 | 1 | 1 |
| 4728 | A07 | 83950 | 2 | 1 |
| 13120880 | D05 | 26271 | 1 | 1 |
| 4762 | D08 | 750690 | 1 | 1 |
| 4751 | F07 | 49842 | 1 | 1 |
| 4734 | D07 | 328403 | 1 | 1 |
| 4731 | C09 | 138389 | 1 | 1 |
| 4749 | G07 | 36354 | 1 | 1 |
| 13120881 | F09 | 376248 | 1 | 1 |
| 13120880 | G02 | 5366 | 1 | 1 |
|  |  |  |  |  |
| **Plate ID** | **Well ID** | **NSC number** | **% viable cells (compound alone)** | **% viable cells (compound + JQ1)** |
| 4751 | G09 | 70929 | 1 | 1 |
| 13120880 | B10 | 87511 | 1 | 1 |
| 4751 | C05 | 353527 | 1 | 1 |
| 13120881 | C10 | 401005 | 1 | 1 |
| 4739 | H08 | 311727 | 1 | 1 |
| 13120881 | B09 | 350085 | 1 | 1 |
| 4743 | D05 | 697726 | 1 | 1 |
| 4723 | B06 | 683648 | 1 | 1 |
| 4743 | B10 | 400978 | 1 | 1 |
| 4734 | F09 | 317003 | 5 | 1 |
| 4734 | D09 | 277184 | 1 | 1 |
| 4734 | H10 | 727038 | 1 | 1 |
| 4734 | H07 | 638432 | 1 | 1 |
| 4733 | C08 | 651084 | 1 | 1 |
| 4746 | H09 | 620358 | 1 | 1 |
| 4751 | C03 | 145366 | 1 | 1 |
| 4724 | H09 | 33353 | 1 | 1 |
| 4734 | A08 | 8675 | 0 | 1 |
| 4739 | C03 | 149286 | 0 | 1 |
| 13120881 | G06 | 305222 | 9 | 1 |
| 4750 | E11 | 30916 | 1 | 0 |
| 4733 | A02 | 637827 | 0 | 0 |
| 4736 | F07 | 122131 | 1 | 0 |
| 4748 | D08 | 168597 | 1 | 0 |
