# Supplementary material for: The BET bromodomain inhibitor exerts the most potent synergistic anticancer effects with quinone-containing compounds and anti-microtubule drugs
Source: Oncotarget. 2016 Oct 13;7(48):79217–32. doi: 10.18632/oncotarget.12640 (PMC5346709; doi:10.18632/oncotarget.12640)
Supplement: Supplementary file 3 [file oncotarget-07-79217-s003.docx]

**Supplementary Dataset S2.** List of compounds which exerted synergistic anticancer effects with JQ1 with combination R values between 0.4 and 0.7 in the secondary screen.

| **Plate** | **Well** | **NSC number** | **Cas number** | **PubChem SID** | **Combination R value** | **Compound name** |
| --- | --- | --- | --- | --- | --- | --- |
| 46 | B6 | 637578 | 118112-10-0 | 581520 92763789 | 0.405 | ANTINEOPLASTIC-637578 or N-[3-(2-Pyridyl)isoquinolin-1-yl]-2-pyridinecarboxamidine |
| 50 | G5 | 328166 | 87532-30-7 | 574668 92763448 | 0.424 | Verrucarin A, 9,10-epoxy-9,10-dihydro-8-hydroxy-, (8.beta.,9.beta.,10.beta.) |
| 51 | G9 | 70929 | 11048-97-8 | 114100 | 0.425 | Hedamycin |
| 63 | D3 | 758252 | 868540-17-4 |  | 0.426 | Carfilzomib or Kyprolis |
| 80 | E7 | 45383 | 3930-19-6 | 99123 26732715 | 0.431 | Streptonigrin, STP, Nigrin, Bruneomycin |
| 45 | B4 | 147340 | 22862-75-5 | 559916 93576890 | 0.439 | Anisomycin hydrochloride or 3,4-Pyrrolidinediol, 2-(p-methoxybenzyl)-, 3-acetate, hydrochloride, cis-2,3,trans-3,4-(-)- |
| 51 | B9 | 325319 | 77327-05-0 | 457933 92763446 | 0.44 | Didemnin B or L-Tyrosine, N-[1-[N-[4-[[3-hydroxy-4-[[N-[N-[1-(2-hydroxy-1-oxopropyl)- L-prolyl]-N-methyl-L-leucyl]-L-threonyl]amino-5-methyl- 1-oxoheptyl]oxy]-2,5-dimethyl-1,3-dioxohexyl]-L-leucyl]- L-prolyl]-N,O-dimethyl-, .phi.-lactone |
| 45 | H8 | 106408 | 5544-25-2 | 406648 | 0.448 | AME, B 89006, Anthramycin methyl ether or 5H-Pyrrolo[2,1-c][1,4]benzodiazepine-2-acrylamide, 1,10,11.beta.,11a.beta.-tetrahydro-9-hydroxy-11-methoxy-5-oxo-, monohydrate or 2-Propenamide, 3-(5,10,11,11a-tetrahydro-9-hydroxy-11-methoxy-8-methyl-5-oxo-1H-pyrrolo[2,1-c][1,4]benzodiazepin-2-yl)-, [11R-(2(E),11.alpha.,11a.beta.)]- |
| 80 | F9 | 85236 | 6754-13-8 | 122585 92763948 | 0.448 | PF 56, Helenalin |
| 48 | F4 | 24818 | 518-28-5 | 86163 92763397 | 0.459 | Podophyllotoxin or Naphtho[2,3-d]-1,3-dioxole-6-carboxylic acid, 5,6,7,8-tetrahydro-8-hydroxy-7-(hydroxymethyl)-5-(3,4,5-trimethoxyphenyl-, .gamma.-lactone |
| 80 | D8 | 60387 | 6879-02-3. | 108410 | 0.471 | Tylocrebin, Lylocrebine |
| 80 | D10 | 94600 | 7689-03-4. | 399733 26732559 | 0.479 | Camptothecin |
| 47 | G7 | 757 | 64-86-8 | 67636 | 0.492 | Colcin, Condylon, Colsaloid, Colchisol, Colchicine, Cholchineos |
| 46 | E5 | 632841 |  | 496043 92763782 | 0.5 | 4-Piperidinone, 1-(1-oxo-2-propenyl)-3,5-bis(phenyl- methylene)- |
| 43 | D11 | 667251 |  | 512196 26665050 | 0.501 | 2-Propenenitrile, 3-[3-(dimethylamino)phenyl]-2-phenyl- |
| 45 | G5 | 352890 | 77691-03-3 | 576189 92763344 | 0.505 | 9-Deazaadenosine or 5H-Pyrrolo[3,2-d]pyrimidine-4-amine, 7-.beta.-D- ribofuranosyl-, monohydrochloride |
| **Plate** | **Well** | **NSC number** | **Cas number** | **PubChem SID** | **Combination R value** | **Compound name** |
| 51 | D7 | 24559 | 18378-89-7 | 85982 | 0.505 | Plicamycin or Mithramycin or Mithracin or Aurelic Acid |
| 81 | B10 | 400978 | 1146-04-9 | 471980 | 0.511 | DR-15977, Illudin M, Illudine M |
| 51 | G3 | 208734 | 57576-44-0 | 125750 | 0.515 | Aclarubicin or Aclacinomycin A or Aclucinomycin A |
| 62 | H6 | 26980 | 50-07-7 | 87663 92763399 | 0.515 | Mitomycin, Mitomycin C, Mutamycin |
| 63 | C2 | 125973 | 33069-62-4 | 418145 | 0.523 | Paclitaxel or Anzatax or Taxol |
| 34 | A8 | 8675 | 2390-59-2 | 539488 92764754 | 0.524 | C.I. 42600, Ethyl Violet |
| 50 | F10 | 690634 |  | 522754 | 0.54 |  |
| 24 | G6 | 105827 | 22242-90-6 | 406267 26666751 | 0.546 | Thiosangivamycin |
| 48 | D8 | 168597 | 13302-14-2 | 441682 | 0.546 | Tributylchlorolead |
| 51 | D10 | 219734 | 57780-57-1 | 129917 | 0.558 | 1,8-Octanediamine, N,N'-di-9-acridinyl- |
| 48 | H4 | 33410 | 63989-75-3 | 91562 92763400 | 0.56 | Colchicinic acid, N-benzoyltrimethyl-, methyl ether |
| 46 | B9 | 323241 | 79514-43-5 | 457681 | 0.563 | 3-Azabicyclo[3.2.2]nonane-3-carboselenoic acid, [1-(2-pyridinyl)ethylidene]hydrazide |
| 51 | H4 | 328426 | 63166-73-4 | 458651 92763449 | 0.565 | Phyllanthoside |
| 50 | F2 | 165563 | 41451-75-6 | 439752 92763420 | 0.568 | Bruceantin or Picras-3-en-21-oic acid, 15-[(3,4-dimethyl-1-oxo-2-pentenyl)oxy]-13,20-epoxy-3,11,12-trihydroxy-2,16-dioxo-, methyl ester [11.beta.,12.alpha.,15.beta.(E]- |
| 34 | H10 | 727038 |  | 48427116 92764524 | 0.571 | CDDO-Im |
| 52 | D3 | 333856 |  | 575022 | 0.571 | Tetrocarcin A, sodium salt or Antlermicin A, monosodium salt |
| 46 | C7 | 44690 | 2150-48-3 | 546540 | 0.574 | Pyronin B, Pyronine B or Ammonium, (6-diethylamino-3H-xanthen-3-ylidene)diethyl-, chloride or Ethanaminium, N-[6-(diethylamino)-3H-xanthen-3-ylidene]-N-ethyl-, chloride |
| 49 | F6 | 689857 |  | 522451 92764423 | 0.575 | Benzoic acid, 4-[[(2,5-dihydroxyphenyl)methyl]amino]-, 1-adamantanemethyl ester |
| 44 | F8 | 63701 | 606-58-6 | 110120 92763404 | 0.576 | B181008, Vengicide, Unamycin B, Toyocamycin, Antibiotic 1037, Antibiotic E 212, 7-Deaza-7-cyanoadenosine, 7H-Pyrrolo[2,3-d]pyrimidine-5-carbonitrile, 4-amino-7-.beta.-D-ribofuranosyl- |
| 47 | E3 | 140377 | 23444-70-4 | 426542 | 0.577 | Arnebin or Alkanin β,β-dimethylacrylate or 2-Butenoic acid, 3-methyl-, 1-(5,8-dihydro- 1,4-dihydroxy-5,8-dioxo-2-naphthalenyl)-4-methyl-3-pentenyl ester |
|  | | | | | | |
| **Plate** | **Well** | **NSC number** | **Cas number** | **PubChem SID** | **Combination R value** | **Compound name** |
| 47 | D6 | 629971 |  | 494811 92763778 | 0.584 | 9-Amino-20-(R,S)-camptothecin or 1H-Pyrano[3',4':6,7]indolizino[1,2-b]quinoline-3,14(4H,12H)- dione, 10-amino-4-ethyl-4-hydroxy-, (R,S)- |
| 51 | H9 | 243023 |  | 568314 | 0.584 | Cinerubin B hydrochloride |
| 44 | D3 | 165897 | 40448-85-9 | 440005 92763421 | 0.586 | 1(2H)Quinolinecarbothioic acid, 2-cyano-6-methoxy-, S-ethyl ester |
| 34 | G10 | 679525 | 106674-03-7 | 518105 92764355 | 0.592 | Benzyl 7-(Methoxycarbonyl)-3,4,5,6-tetrahydro-4,5-dioxo- 6-((4-methylphenyl)sulfonyl)benzo[1,2-b:4,3-b']dipyrrole-1-carboxylate |
| 62 | H10 | 82151 | 23541-50-6 | 301154 | 0.596 | Daunorubicin hydrochloride or Daunomycin hydrochloride or Rubidomycin hydrochloride |
| 45 | E7 | 667467 |  | 512260 | 0.6 | 1,4-Thiazino[3,2-c]quinoline-3-thione, 2-phenyl- |
| 50 | A3 | 172924 | 14907-98-3 | 444366 92764050 | 0.6 | Brusatol |
| 50 | G11 | 52141 | 6833-84-7 | 103268 | 0.602 | Nonactin |
| 62 | F10 | 747973 | 219989-84-1 |  | 0.603 | Ixabepilone or Ixempra or Azaepothilone B |
| 63 | B2 | 761432 | 183133-96-2 |  | 0.604 | Cabazitaxel or Jevtana |
| 45 | H11 | 651079 |  | 505177 | 0.611 | ANTINEOPLASTIC-651079 or 1H-Pyrrolo[1,2-a]benzimidazole-5,8-dione, 6-(1-aziridinyl)- 2,3-dihydro-3-(propionyl)-7-methyl |
| 43 | A7 | 65423 | 550-33-4 | 110786 | 0.63 | Nebularine, Purinosine, Purine riboside |
| 43 | B10 | 400978 | 1146-04-9 | 471980 | 0.633 | Illudin M, Illudine M, Spiro[cyclopropane-1,5'-[5H]inden]-7'(6'H)-one, 2',3'-dihydro-3',6'-dihydroxy-2',2',4',6'-tetramethyl-,(3'S-trans)- |
| 44 | A3 | 145669 | 31785-60-1 | 428980 92764031 | 0.634 | 4(1H)-Quinazolinone, 2,3-dihydro-2-(1-naphthalenyl)- |
| 47 | C10 | 403148 | 19186-35-7 | 473592 92763355 | 0.647 | Anthricin, Silicicolin, (-)-Anthricin, 4-Deoxypodophyllotoxin or Furo[3',4':6,7]naphtho[2,3-d]-1,3-dioxol-6(5aH)-one, 5,8,8a,9-tetrahydro-5-(3,4,5-trimethoxyphenyl)-,(5R,5aR,8aR)- |
| 48 | E3 | 687849 |  | 521761 92764376 | 0.653 |  |
| 47 | A8 | 24817 | 568-53-6 | 86162 | 0.654 | Peltatin A (or α) or Naphtho[2,3-d]-1,3-dioxole-6-carboxylic acid, 5,6,7,8-tetrahydro-9-hydroxy-5-(4-hydroxy-3,5-dimethoxyphenyl)-7-(hydroxymethyl)-, .gamma.-lactone |

| **Plate** | **Well** | **NSC number** | **Cas number** | **PubChem SID** | **Combination R value** | **Compound name** |
| --- | --- | --- | --- | --- | --- | --- |
| 49 | G8 | 126728 | 3148-09-2. | 92763411 | 0.656 | 379Y, Verrucarin A, Muconomycin A, Antibiotic 379Y or Spiro(16,18-methano-1H,3H,23H-[1,6,12]trioxacyclooctadecino[3,4-d][1]benzopyran-17(18H),2'-oxirane)-3,9,14-trione, 4,5,6,7,16,16a,19a,22-octahydro-4-hydroxy-5,16a,21-trimethyl-, stereoisomer |
| 51 | G7 | 58514 | 7059-24-7 | 548532 | 0.683 | Toyomycin or Chromomycin or Aburamycin B or Olivomycin D |
| 51 | F10 | 700582 |  | 527076 | 0.689 | [1,2,3,4]tetrathiocino[5,6-d:7,8-d']diimidazole -2,9-dithione, 1,3,8,10-tetrabutyl-1,2,3,8,9,10-hexahydro- |
| 50 | H4 | 269754 |  | 570347 92763437 | 0.689 | Modified new trichothecene or Verrucarin A, 7'-deoxy-2'-deoxy-4',8-dihydroxy-7'-(1-hydroxyethyl)- |
| 48 | G4 | 24819 | 518-29-6 | 86164 92763398 | 0.692 | Peltatin β or Naphtho[2,3-d]-1,3-dioxole-6-carboxylic acid, 5,6,7,8-tetrahydro-9-hydroxy-7-(hydroxymethyl)-5-(3,4,5-trimethoxyphenyl)-, .gamma.-lactone |
