# Supplementary material for: The BET bromodomain inhibitor exerts the most potent synergistic anticancer effects with quinone-containing compounds and anti-microtubule drugs
Source: Oncotarget. 2016 Oct 13;7(48):79217–32. doi: 10.18632/oncotarget.12640 (PMC5346709; doi:10.18632/oncotarget.12640)
Supplement: Supplementary file 4 [file oncotarget-07-79217-s004.docx]

**Supplementary Dataset S3.** List of compounds which exerted strong synergistic anticancer effects with JQ1 with combination R values less than 0.4 in the secondary screen.

| **Plate** | **Well** | **NSC number** | **Cas number** | **PubChem SID** | **Combination R value** | **Compound name** |
| --- | --- | --- | --- | --- | --- | --- |
| 50 | D4 | 258812 | 70095-84-0 | 569428 92763379 | 0.061 | N,N-Dimethyldaunomycin hydrochloride or 5,12-Naphthacenedione, 8-acetyl-7,8,9,10-tetrahydro-6,8,11-trihydroxy-1-methoxy-10-[[2,3,6-trideoxy-3-(dimethylamino)-.alpha.-L-lyxo-hexopyranosyl]oxy]-,hydrochloride |
| 50 | E9 | 659999 | Not available | 509287 92764784 | 0.094 | Naphthalene-1,4-dione, 2-bromo-5,8-dihydroxy- 3-[4-[2-(2-hydroxyethoxy)ethyl]-1-piperazinyl]-, hydrochloride |
| 51 | B2 | 70845 | 1404-15-5 | 114031 | 0.111 | Nogalamycin or 2,6-Epoxy-2H-naphthaceno[1,2-b]oxocin-14-carboxylic acid, 11-[(6-deoxy-3-C-methyl-2,3,4-tri-O-methyl-.alpha.-L-mannopyranosyl)oxy]-4-(dimethylamino)-3,4,5,6,9.11,12,13,14,16-decahydro-3,5,8,10,13-pentahydroxy-6,13-dimethyl-9,16-dioxo- |
| 51 | H7 | 67574 | 2068-78-2 | 112141 92764772 | 0.145 | Vincristine sulfate or Onkovin or Leurocristine sulfate or Vincasar or Vincrex or Kyocristine |
| 62 | A8 | 681239 | 25316-40-9 | 301181 | 0.2 | Bortezomib or Velcade |
| 80 | D11 | 122023 | 2001-95-8 | 556534 | 0.225 | Valinomycin, Valinomicin |
| 28 | A7 | 83950 | 3930-19-6 | 121832 26732704 | 0.241 | Streptonigrin, Nigrin, Bruneomycin |
| 51 | G6 | 669356 | Not available | 513140 92764826 | 0.243 | L-Prolinamide, N,N-dimethyl-L-valyl-L-valyl-N-methyl- L-valyl-L-prolyl-N-(phenylmethyl)-, hydrochloride |
| 51 | D3 | 153858 | 35846-53-8 | 92763419 | 0.246 | MTS or Maysanine or Maytansin or Maitansine or Alanine, N-acetyl-N-methyl-, 6-ester with 11-chloro-6,21-dihydroxy-12,20-dimethoxy-2,5,9,16-tetramethyl-4,24-dioxa-9,22-diazatetracyclo[19.3.1.1(10,24).0(3,5)]hexacosa-10,12,14[26],16,18-pentaene-8,23-dione |
| 52 | F2 | 3053 | 50-76-0 | 538571 | 0.273 | Dactinomycin or Cosmegen or Actinomycin D or Oncostatin K or Meractinomycin |
| 51 | E10 | 243928 | 59988-01-1 | 135643 | 0.281 | Ethanesulfonamide, N-[4-(9-acridinylamino)-3-methoxyphenyl]-, monomethanesulfonate |
| 62 | G10 | 754143 | 128517-07-7 | 131408687 | 0.29 | Depsipeptide or Romidepsin |
| 81 | F7 | 332598 | 90996-54-6 | 459478 92763340 | 0.293 | Rhizoxin |
| 81 | C2 | 131547 | 2632-29-3 | 421449 92763412 | 0.306 | Marckine, Tubolosine, Tubulosine |
| 45 | E5 | 330770 | 82585-91-9 | 459104 92763849 | 0.306 | Carbamic acid, (5-amino-1,2-dihydro-3-phenylpyrido[3,4-b]pyrazin-7-yl)-, ethyl ester |
| **Plate** | **Well** | **NSC number** | **Cas number** | **PubChem SID** | **Combination R value** | **Compound name** |
| 48 | F10 | 349644 | 82423-05-0 | 92763343 | 0.31 | Cyanocycline A or 4,6-Methano-5H-benz[h]oxazolo[3,2-a]pyrazino[3,2,1-de][1,5]naphthyridine-7-carbonitrile, 1,2,3a,4,4a,6,7,9,10,13,13b,13c-dodecahydro-9-(hydroxymethyl)-11-methoxy-5,12-dimethyl-10,13-dioxo- |
| 81 | C4 | 210236 | Not available | 126104 92764120 | 0.311 | Crassin |
| 50 | E11 | 30916 | Not available | 543513 | 0.311 | MP 317, SKI23340 or P-Dioxane, 2,5-bis[(benzoyloxymercuri)methyl]- |
| 47 | E8 | 126727 | 32970-80-2 | 557208 | 0.316 | DESMETHOXY-β-PELTATIN-A METHYL ETHER |
| 81 | F10 | 526417 | 512-64-1 | 580231 92763358 | 0.337 | Levomycin, Echinomycin, Quinomycin A |
| 49 | E8 | 102815 | Not available | 405113 | 0.34 | 7-O-Methylnogalarol or Daunomycin compound D or Nogalarol, 7-O-methyl- |
| 43 | D5 | 697726 | Not available | 525640 92763376 | 0.34 | RH-1 or 2,5-Diaziridinyl-3-(hydroxymethyl)- 6-methyl-1,4-benzoquinone |
| 63 | A2 | 628503 | 114977-28-5 | 494081 | 0.342 | Docetaxel |
| 46 | G5 | 635121 | Not available | 497451 | 0.351 | N'-[(1Z)-1-(1,4-benzothiazin-2-ylidene)ethyl]-2-hydroxybenzohydrazide |
| 49 | E3 | 639828 | 134742-26-0 | 499861 92763366 | 0.352 | Antineoplastic 639828 or Benzamide, 2-amino-N-[[[4-[(5-bromo-2-pyrimidinyl)oxy]- 3-chlorophenyl]amino]carbonyl]- |
| 63 | C3 | 608210 |  | 485117 | 0.359 | Vinorelbine tartrate or Navelbine or Biovelbin |
| 45 | E4 | 175636 | 34934-08-2 | 445485 92764056 | 0.362 | 4(1H)-Quinazolinone, 6-chloro-2,3-dihydro-2-(1-naphthalenyl)- |
| 46 | H9 | 620358 |  | 489740 92763825 | 0.373 | Methyl 13-hydroxy-15-oxo-kaurenoate |
| 51 | F7 | 49842 | 143-67-9 | 101900 | 0.38 | Vinblastine Sulfate Hydrate or Velban or Belvan or Exal or Velbe |
| 34 | F10 | 654260 |  | 506267 92764780 | 0.394 | L-alanylaminophenylbenzoylurea |
| 51 | C4 | 265450 |  | 139837 92764134 | 0.395 | Nogamycin or Disnogamycin or Nogalomycin C or 2,6-Epoxy-2H-naphthaceno[1,2-b]oxocin-9,16-dione, 11-[(6- deoxy 3-C-methyl-2,3,4-tri-O-methyl-.alpha.-L-manno pyranosyl)oxy]-4-(dimethylamino)- 3,4,5,6,11,12,13,14-octahydro-3,5,8,10,13-pentahydroxy- 6,13-dimethyl- |
| 50 | A2 | 129414 | 14358-43-1 |  | 0.396 | (+-)-2,3-Dehydroemetine dihydrochloride |
| 45 | B5 | 267461 | 52934-83-5 | 140408 92764137 | 0.398 | Nanaomycin, Nanomycin A |
